# SERPINB3-MYC axis induces the basal-like/squamous subtype and enhances disease progression in pancreatic cancer

**DOI:** 10.1016/j.celrep.2023.113434

**Published:** 2023-11-18

**Authors:** Yuuki Ohara, Wei Tang, Huaitian Liu, Shouhui Yang, Tiffany H. Dorsey, Helen Cawley, Paloma Moreno, Raj Chari, Mary R. Guest, Azadeh Azizian, Jochen Gaedcke, Michael Ghadimi, Nader Hanna, Stefan Ambs, S. Perwez Hussain

**Affiliations:** 1 Pancreatic Cancer Section, Laboratory of Human Carcinogenesis, Center for Cancer Research, National Cancer Institute, National Institutes of Health, Bethesda, MD 20892, USA; 2 Laboratory of Human Carcinogenesis, Center for Cancer Research, National Cancer Institute, National Institutes of Health, Bethesda, MD 20892, USA; 3 Genome Modification Core, Laboratory Animal Sciences Program, Frederick National Lab for Cancer Research, Frederick, MD 21701, USA; 4 Städtisches Klinikum Karlsruhe, Moltkestraße 90, 76133 Karlsruhe, Germany; 5 Department of General, Visceral and Pediatric Surgery, University Medical Center Göttingen, Robert-Koch-Straße 40, 37075 Göttingen, Germany; 6 Division of General & Oncologic Surgery, University of Maryland School of Medicine, Baltimore, MD 21201, USA; 7 Data Science & Artificial Intelligence, R&D, AstraZeneca, Gaithersburg, MD 20878, USA; 8 Lead contact

## Abstract

Pancreatic ductal adenocarcinoma (PDAC) exhibits distinct molecular subtypes: classical/progenitor and basal-like/squamous. Our study aimed to identify genes contributing to the development of the basal-like/squamous subtype, known for its aggressiveness. Transcriptome analyses revealed consistent upregulation of SERPINB3 in basal-like/squamous PDAC, correlating with reduced patient survival. *SERPINB3* transgene expression in PDAC cells enhanced *in vitro* invasion and promoted lung metastasis in a mouse PDAC xenograft model. Metabolome analyses unveiled a metabolic signature linked to both SERPINB3 and the basal-like/squamous subtype, characterized by heightened carnitine/acylcarnitine and amino acid metabolism, associated with poor prognosis in patients with PDAC and elevated cellular invasiveness. Further analysis uncovered that SERPINB3 inhibited the cysteine protease calpain, a key enzyme in the MYC degradation pathway, and drove basal-like/squamous subtype and associated metabolic reprogramming through MYC activation. Our findings indicate that the SERPINB3-MYC axis induces the basal-like/squamous subtype, proposing SERPINB3 as a potential diagnostic and therapeutic target for this variant.

## In brief

Ohara et al. use a comprehensive transcriptome and metabolome analysis to show that the SERPINB3-MYC axis drives the aggressive basal-like/squamous molecular subtype in pancreatic ductal adenocarcinoma. This axis also promotes subtype-associated metabolic alterations, including heightened carnitine/acylcarnitine and amino acid metabolism, correlating with poorer patient prognosis.

## Graphical Abstract

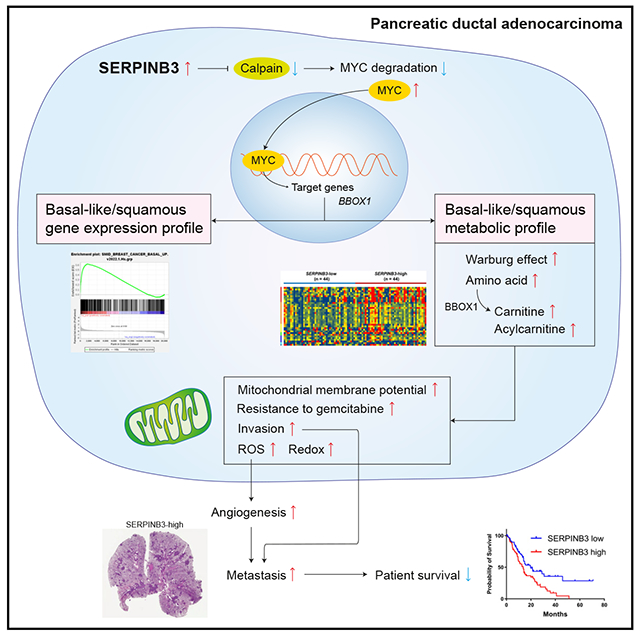

## INTRODUCTION

Pancreatic cancer is a lethal cancer with a 5 year survival of only 12%.^1^ Pancreatic ductal adenocarcinoma (PDAC) is the most common histologic form and comprises more than 90% of all malignancies in the pancreas.^2^

Earlier studies identified several molecular subtypes of PDAC with differences in biological features and patient survival.^3–5^ Moffitt et al. proposed that PDAC can be separated into two subtypes: the basal-like and the classical subtype.^4^ The basal-like subtype has characteristics similar to those of basal tumors in breast and bladder cancer. They also classified the stroma into normal and activated. In contrast, Bailey et al. argued that PDAC can be classified into four subtypes: squamous, immunogenic, pancreatic progenitor, and aberrantly differentiated endocrine exocrine.^3^ The squamous subtype was associated with the most aggressive disease and exhibited distinct features, including metabolic reprogramming, activated MYC pathways, squamous differentiation, inflammation, and hypoxia.^3^ Recently, a comprehensive molecular subtype analysis classified PDAC into two subtypes: classical/progenitor and basal-like/squamous.^6^ The classical/progenitor subtype possesses an endodermal-pancreatic characteristic, while the basal-like/squamous subtype has lost this identity.^7^

Metabolic reprogramming is reported as one of the hallmarks of cancer.^8^ Previous studies have described metabolic adaptations in PDAC as one of the key events in growth and progression.^8,9^ Cancer cells increase aerobic glycolysis, amino acid and lipid synthesis, and macromolecule synthesis to fulfill the high demand of energy and regulate oxidative stress.^8–11^ In terms of metabolic adaptations, PDAC subtypes may have distinct metabolic profiles.^12–16^ The classical/progenitor subtype relies on lipid metabolism/fatty acid oxidation for its metabolic needs, whereas the basal-like/squamous subtype is more dependent on glycolysis.^12–16^ Yet, the relationship between the metabolic and the gene expression profiles has not been well elucidated for PDAC. Most of the previous studies were based on cell lines or a small number of PDAC patient samples. Using a large PDAC patient cohort and human PDAC cell lines, we hypothesized that an integrative transcriptome and metabolome approach may identify potential contributors to the development of the aggressive basal-like/squamous subtype.

To test this hypothesis, we initially analyzed two large datasets (GSE36924^3^ and GSE71729^4^) to filter the genes commonly associated with the basal-like/squamous subtype in both datasets. Subsequently, we validated these candidate genes in our NCI-UMD-German cohort for their association with patient survival. Furthermore, these genes were examined for their expression levels in tumors and the adjacent nontumor pancreas in our NCI-UMD-German cohort. This integrated analysis resulted in the identification of serine/cysteine protease inhibitor family B member 3 (SERPINB3; also known as squamous cell carcinoma antigen 1, SCCA1) as a top candidate gene associated with poor patient survival in the basal-like/squamous subtype. Hence, we selected it for further investigation of its functional role in disease progression and confirmed its involvement in the basal-like/squamous differentiation of PDAC.

## RESULTS

### SERPINB3 is upregulated in the basal-like/squamous subtype of PDAC and associates with poor survival

**Figure 1.**
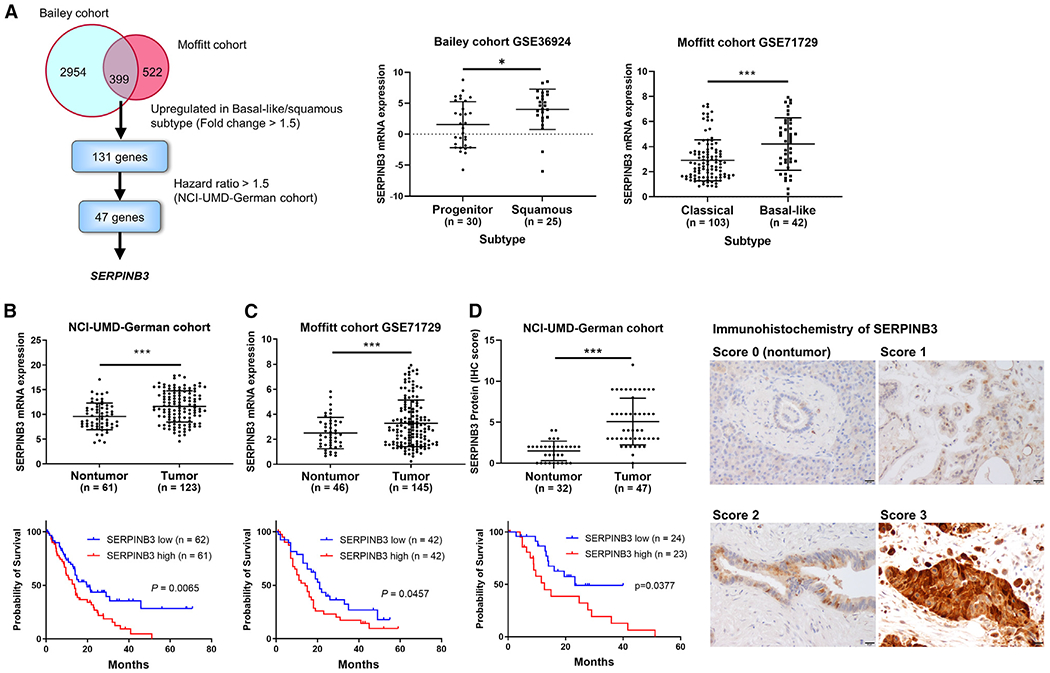
Transcriptome analysis identifies SERPINB3 as a marker upregulated in basal-like/squamous PDAC and associated with poor survival (A) Strategy to find candidate driver genes of the basal-like/squamous subtype in PDAC. Two cohorts (Bailey^3^ and Moffitt^4^) were interrogated to identify candidate driver genes of the basal-like/squamous subtype. The genes were narrowed down to those associated with patient survival in the NCI-UMD-German cohort.^17^ Data are presented as the mean ± SD. *p < 0.05, ***p < 0.005 by unpaired two-tailed Student’s t test. (B–D) Comparison of SERPINB3 transcript and protein expression levels in PDAC tumors versus adjacent noncancerous tissues, revealing upregulation of SERPINB3 in tumors in both the NCI-UMD-German cohort (mRNA via qPCR and protein via IHC) and the validation cohort (Moffitt cohort^4^; GSE71729; mRNA). Data are presented as the mean ± SD. ***p < 0.005 by unpaired two-tailed Student’s t test. The bottom shows Kaplan-Meier plots and log-rank test results, highlighting the association of increased SERPINB3 expression with decreased PDAC patient survival. For the survival analysis in the NCI-UMD-German cohort (B and D), the comparison was conducted between patients in the upper and lower 50% of SERPINB3 levels. In the validation cohort (C), patients in the upper and lower tertiles of *SERPINB3* expression were compared (total patient number = 125). The right side shows IHC of SERPINB3 in representative nontumor (score 0) and tumor tissues (scores 1–3). SERPINB3 protein was detected in both the nucleus and the cytoplasm, as shown by the brown DAB-based IHC in the tumor cells. The images show the staining strength (score 0, unstained; score 1, weak; score 2, moderate; score 3, strong). Scale bars, 20 μm. More details can be found in the STAR Methods. IHC, immunohistochemistry. See also Tables S1 and S2.

Our study aimed to identify candidate driver genes involved in the development and progression of the basal-like/squamous subtype of PDAC. We initially analyzed transcriptome data from two PDAC cohorts (Bailey cohort^3^ and Moffitt cohort^4^) and identified 399 genes that were differentially expressed in the basal-like/squamous subtype compared with the classical/progenitor subtype (cutoffs: fold change > 1.5 or < −1.5, p < 0.05), with 131 of the genes being upregulated in the basal-like/squamous subtype (Figure 1A). The genes were then interrogated for their association with patient survival (thresholds: hazard ratio > 1.5 using Cox regression analysis, p < 0.05) in our NCI-UMD-German patient cohort,^17^ yielding a narrowed list of 47 genes (Table S1). Among them, *SERPINB3* was ranked one of the top and robustly upregulated genes in the basal-like/squamous subtype (Table S1). SERPINB3 is also known as squamous cell carcinoma antigen,^18^ associates with hypoxia,^19,20^ and promotes MYC activation,^21,22^ cancer stemness,^23,24^ metabolic reprogramming,^19^ and inflammation.^25–27^ These functions overlap with the characteristics of the basal-like/squamous subtype^3^; thus, we chose SERPINB3 for further investigation. *SERPINB3* transcript levels were upregulated in tumor tissues compared with nontumor tissues, and the upregulation of SERPINB3 in PDAC was associated with decreased patient survival in both the NCI-UMD-German cohort and a validation cohort (Moffitt cohort^4^) (Figures 1B and 1C). Notably, *SERPINB3* transcripts were undetectable in about one-third (31/92) of the nontumor samples but measurable in 97% (123/127) of our NCI-UMD-German cohort patient tumors using quantitative real-time PCR assay (qPCR) (Figure 1B). Furthermore, SERPINB3 protein was detected in both the nucleus and the cytoplasm of tumor cells by immunohistochemistry (IHC) (Figure 1D). In addition, SERPINB3 protein upregulation in tumors also associated with a decreased survival in our NCI-UMD-German patient cohort (Figure 1D; Table S2), consistent with the aforementioned association of upregulated *SERPINB3* transcript expression with decreased patient survival. These findings support the hypothesis that SERPINB3 is an oncogene whose expression promotes the aggressive basal-like/squamous subtype of PDAC, leading to decreased survival of patients with tumors exhibiting high SERPINB3 expression.

### Upregulation of SERPINB3 induces characteristics of the basal-like/squamous subtype of PDAC and promotes metastasis in an orthotopic mouse model

**Figure 2.**
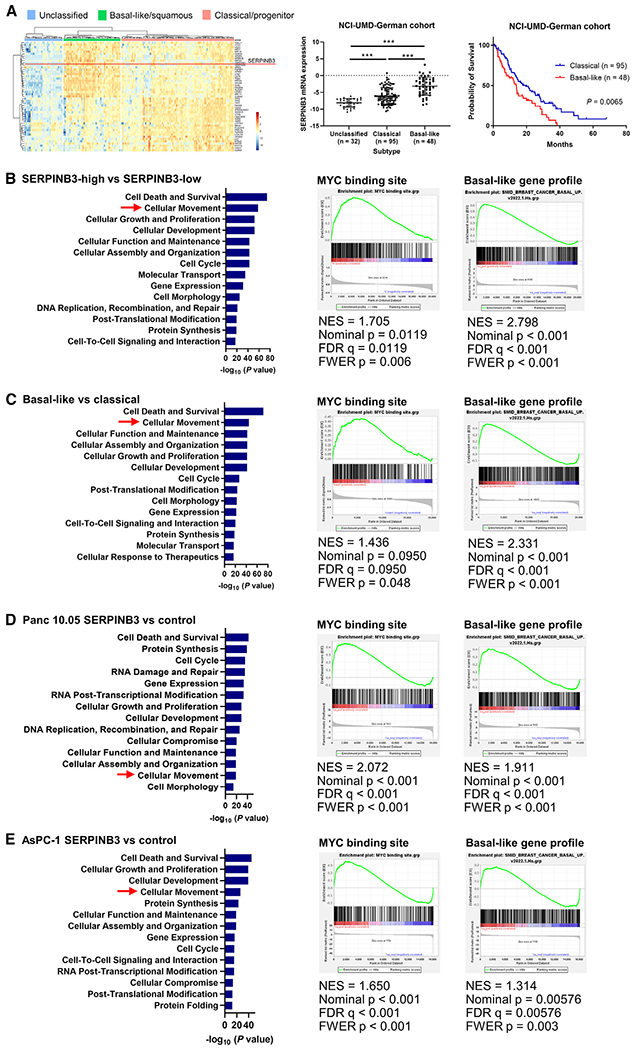
Upregulation of SERPINB3 promotes the characteristics of the basal-like/squamous subtype of PDAC (A) The transcriptome of PDAC defines its molecular subtypes. Previously described gene signatures for molecular subtypes^4^ separated 175 patients of our NCI-UMD-German cohort into unclassified (n = 32), basal-like/squamous (n = 48), and classical/progenitor (n = 95) subtypes. In the middle, tumors defined as basal-like/squamous show upregulation of *SERPINB3* mRNA expression. Data are presented as the mean ± SD; ***p < 0.005 by one-way ANOVA. On the right, the Kaplan-Meier plot and log-rank test show the reduced survival of patients with basal-like/squamous PDAC. (B–E) IPA (blue bars) and GSEA show similar pathway patterns, including enrichment of cellular movement, MYC activation,^28,29^ and induction of the basal-like gene expression profile,^30^ in *SERPINB3*-high and basal-like/squamous PDAC tumors (B and C) and in human PDAC cell lines with *SERPINB3* transgene overexpression (D and E). *SERPINB3*-low, n = 87, and *SERPINB3*-high, n = 88 in (B). Classical/progenitor subtype, n = 95, and basal-like/squamous subtype, n = 48 in (C). Panc 10.05 control, n = 4, and Panc 10.05 SERPINB3, n = 4 in (D). AsPC-1 control, n = 4, and AsPC-1 SERPINB3, n = 4 in (E). IPA, Ingenuity pathway analysis; GSEA, gene set enrichment analysis. See also Figure S1.

Next, we investigated whether SERPINB3 expression promotes basal-like/squamous subtype characteristics with enhanced disease aggressiveness. Previously described gene signatures for PDAC molecular subtypes^4^ separated 175 patients of our NCI-UMD-German cohort into unclassified, basal-like/squamous, and classical/progenitor subtypes (Figure 2A). Tumors defined as the basal-like/squamous subtype showed upregulation of *SERPINB3* mRNA expression and were associated with worse patient survival (Figure 2A). In the pathway enrichment analysis using the Ingenuity pathway analysis (IPA) in the NCI-UMD-German cohort, both the *SERPINB3*-high and basal-like/squamous PDAC tumors showed similar pathway enrichment patterns, including enrichment of cellular movement (Figures 2B and 2C). Additional gene set enrichment analysis (GSEA) revealed the significant upregulation of 374 MYC signature genes,^28,29^ defined by their MYC binding sites, and a basal-like gene expression profile^30^ derived from human breast tumors, in the transcriptome of *SERPINB3*-high and basal-like/squamous PDAC tumors (see enrichment plots in Figures 2B and 2C).

**Figure 3.**
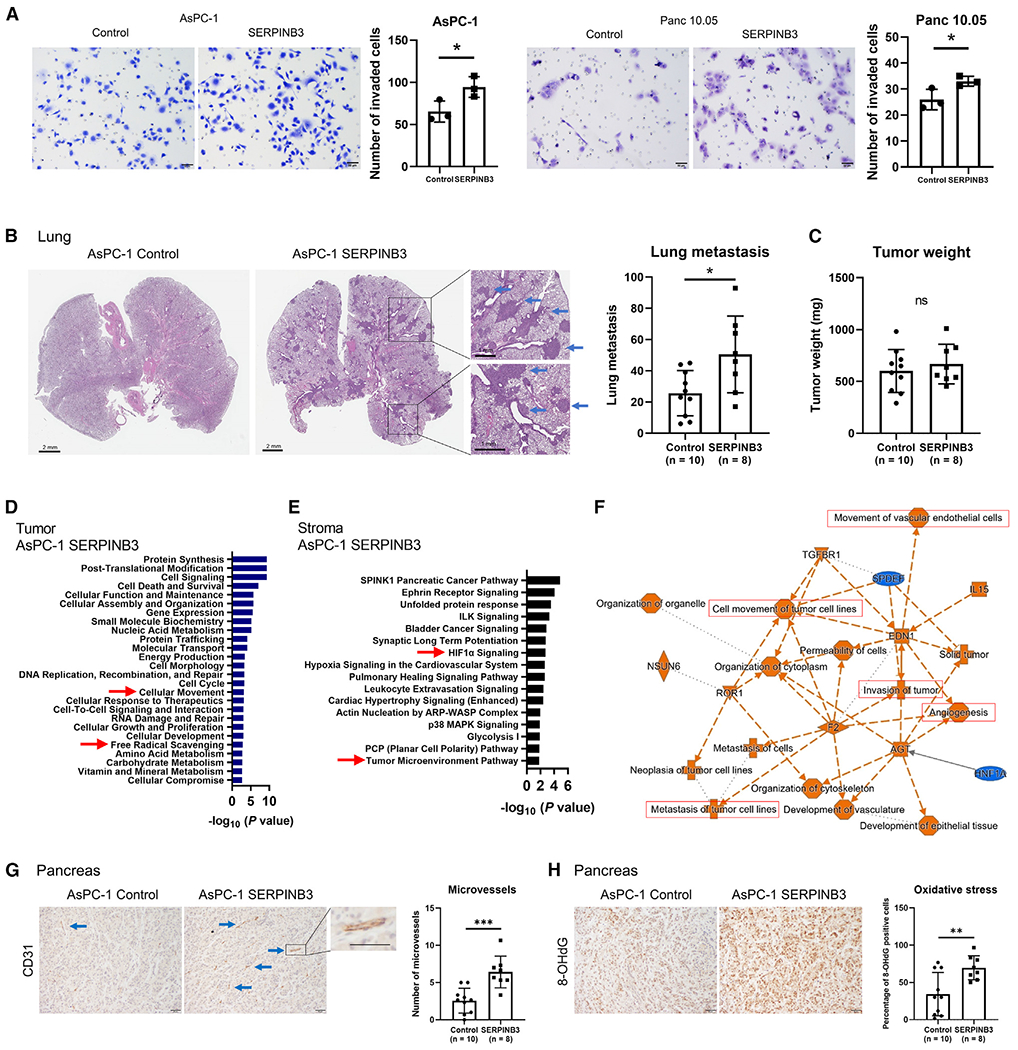
Upregulation of SERPINB3 promotes invasion and metastasis of PDAC *SERPINB3* transgene-expressing PDAC cells were examined to define the function of SERPINB3. (A) SERPINB3-overexpressing PDAC cells demonstrate an increased invasive ability. Cells that passed through a cell culture insert membrane coated with Matrigel were fixed, and the cells were counted. Data are presented as the mean ± SD of three independent experiments; *p < 0.05 by unpaired two-tailed Student’s t test. (B–H) Vector control and SERPINB3-overexpressing AsPC-1 human PDAC cells were transplanted into the pancreas of immune-deficient (NOD-SCID) mice. The mice were euthanized at 5 weeks, and both tumor burden and lung metastases were assessed. (B) The increased number of lung metastases in mice transplanted with AsPC-1 cells carrying the *SERPINB3* transgene (shown in the graph to the right). Basophilic clusters indicate the metastatic lesions in sections of the lung (see arrows). Data are presented as the mean ± SD (AsPC-1 control, n = 10 mice; AsPC-1 SERPINB3, n = 8 mice); *p < 0.05 by unpaired two-tailed Student’s t test. (C) Upregulation of SERPINB3 does not increase the weight of the primary tumor xenografts in the pancreas. Data are presented as the mean ± SD (AsPC-1 control, n = 10 mice; AsPC-1 SERPINB3, n = 8 mice); ns, not significant by unpaired two-tailed Student’s t test. (D) Pathway enrichment analysis using IPA for the primary tumor xenografts from SERPINB3-overexpressing AsPC-1 versus vector control cells. IPA indicates the activation of a set of pathways, including “cellular movement” and “free radical scavenging,” in SERPINB3-overexpressing AsPC-1, consistent with increased invasion (A) and oxidative stress (Figure S2) in these tumors. (E and F) Pathway enrichment analysis using IPA for the tumor stroma from xenografts of SERPINB3-overexpressing AsPC-1 versus vector control cells. The stroma in AsPC-1 SERPINB3 xenografts exhibits activation of pathways associated with “HIF1α” and “tumor microenvironment,” including “angiogenesis” and “metastasis/invasion/cell movement.” (G) The density of microvessels (angiogenesis) in the tumor xenografts examined by IHC for CD31. Microvessel density is increased in the stroma of SERPINB3-overexpressing AsPC-1 xenografts (arrows). Scale bars, 50 μm. Data are presented as the mean ± SD (AsPC-1 control, n = 10 mice; AsPC-1 SERPINB3, n = 8 mice); ***p < 0.005 by unpaired two-tailed Student’s t test. (H) IHC for 8-OHdG, a marker of oxidative stress, in the tumor xenografts. The percentage of 8-OHdG-positive cells, indicating the level of oxidative stress, was assessed. The level of oxidative stress is increased in SERPINB3-overexpressing AsPC-1 xenografts. Scale bars, 50 μm. Data are presented as the mean ± SD (AsPC-1 control, n = 10 mice; AsPC-1 SERPINB3, n = 8 mice); **p < 0.01 by unpaired two-tailed Student’s t test. 8-OHdG, 8-hydroxy-2′-deoxyguanosine; IHC, immunohistochemistry; IPA, Ingenuity pathway analysis. See also Figure S2.

To follow up on our observations in patient tumors, we examined SERPINB3 expression levels in several human PDAC cell lines. Most of them expressed SERPINB3 at detectable levels (Figure S1A). AsPC-1, MIA PaCa-2, and Panc 10.05 cells were then selected to establish cell lines with *SERPINB3* transgene overexpression. These cell lines are of the classical/progenitor PDAC subtype^31^ and can therefore be used to investigate the hypothesis that SERPINB3 overexpression induces the transition of the classical/progenitor subtype into the basal-like/squamous subtype. SERPINB3 overexpression was confirmed at the protein level in these cell lines (Figure S1B). Pathway enrichment analyses using IPA and GSEA indicated a coherent activation of the same pathways across these human PDAC cell lines with *SERPINB3* transgene expression (Figures 2D and 2E), mirroring the findings from the *SERPINB3*-high and basal-like/squamous PDAC tumors. Because MYC protein is known to be rapidly degraded, previous studies investigated and showed that enhanced MYC signaling is reliably examined by assessing downstream gene expression, such as MYC binding site analysis through GSEA.^28,29^ Nevertheless, for SERPINB3-overexpressing Panc 10.05 cells, we observed a persistent upregulation of MYC protein (Figure S1C). To elucidate SERPINB3 functions, additional *in vitro* experiments were performed using SERPINB3-overexpressing PDAC cells. SERPINB3 did not have obvious effects on proliferation (Figure S2A). In contrast, SERPINB3 overexpression significantly enhanced the invasive ability of PDAC cell lines and reduced the sensitivity to the standard-of-care drug, gemcitabine (Figures 3A and S2B).

To further investigate the mechanistic and functional role of SERPINB3 in tumor progression, we utilized an orthotopic PDAC mouse model. The implantation of SERPINB3-overexpressing AsPC-1 cells in the pancreas of mice led to a significant enhancement in lung metastases (Figure 3B). However, upregulation of SERPINB3 did not alter primary tumor growth of AsPC-1 cells, as indicated by the comparable tumor weights in the SERPINB3-expressing and control cells (Figure 3C). Furthermore, RNA-sequencing transcriptome and pathway analysis of the primary tumor xenografts revealed that upregulated SERPINB3 enhanced cellular movement and free radical scavenging (Figure 3D), which is consistent with our observations from patient tumors and cultured cells indicating an increased invasion ability (Figure 3A) and oxidative stress (Figure S2C) in the presence of upregulated SERPINB3. IPA for the xenograft stroma revealed the activation of pathways associated with HIF1α signaling, movement of tumor cells and endothelial cells, and angiogenesis (Figures 3E and 3F). Furthermore, IHC of the endothelial marker CD31 and the reactive oxygen species (ROS) marker 8-hydroxy-2′-deoxyguanosine (8-OHdG) revealed that angiogenesis and oxidative stress are upregulated in the tumor xenografts of SERPINB3-overexpressing AsPC-1 in the orthotopic model (Figures 3G and 3H). SERPINB3 expression in AsPC-1 cells was maintained in both the primary lesions and the lung metastases (Figure S2D). These findings reveal that SERPINB3 upregulation in PDAC enhances the tumor metastatic potential *in vivo.*

### SERPINB3-MYC signaling maintains the basal-like/squamous subtype in PDAC

**Figure 4.**
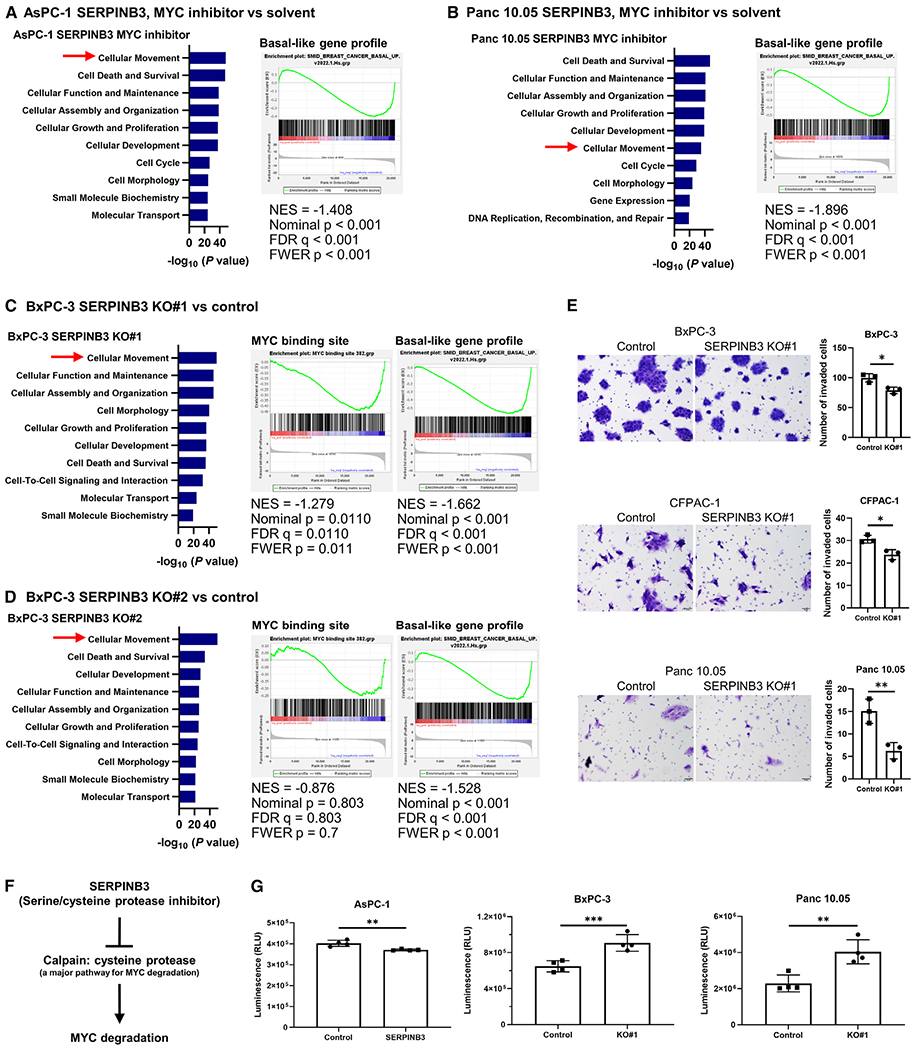
SERPINB3 knockout and inhibition of MYC signaling abrogates the differentiation into the basal-like/squamous subtype of PDAC (A–D) IPA (blue bars) and GSEA indicate similar pathway patterns following either treatment with a MYC inhibitor (10058-F4, 100 μM) (A and B) or SERPINB3 knockout (C and D), including enrichment of cellular movement and decreased basal-like/squamous differentiation, mirroring the observations made in SERPINB3-expressing PDAC cells as well as *SERPINB3*-high and basal-like/squamous PDAC tumors (Figure 2). (E) SERPINB3 knockout decreases the ability of PDAC cells to invade. Cells that passed through a cell culture insert membrane coated with Matrigel were fixed, and the cells were counted. Data are presented as the mean ± SD of three independent experiments; *p < 0.05, **p < 0.01 by unpaired two-tailed Student’s t test. (F) Calpain, a cysteine protease, is a key pathway for MYC degradation.^32^ (G) Calpain activity was reduced in the SERPINB3-overexpressing PDAC cell line (AsPC-1) but activated in SERPINB3-knockout PDAC cell lines (BxPC-3 and Panc 10.05). Data are presented as the mean ± SD (n = 4 for each group); **p < 0.01, ***p < 0.005 by unpaired two-tailed Student’s t test. IPA, Ingenuity pathway analysis; GSEA, gene set enrichment analysis. See also Figure S3.

To further explore the function of SERPINB3 and MYC signaling in the development and maintenance of the basal-like/squamous subtype in PDAC, we established SERPINB3-knockout PDAC cell lines using CRISPR-Cas9 technology and cultured *SERPINB3* transgene-expressing PDAC cells in the presence of a MYC inhibitor (10058-F4). BxPC-3 and CFPAC-1 cell lines, which naturally express relatively high levels of SERPINB3 (Figure S1A) and represent basal-like/squamous PDAC,^31^ were selected to establish SERPINB3-knockout cell lines. The Panc 10.05 cell line, representing the classical/progenitor subtype,^31^ was also selected. Successful SERPINB3 editing was confirmed at the mRNA, protein, and DNA levels within these cell lines (Figures S3A–S3E). When studying the effects of SERPINB3 knockout and MYC inhibition, we noted that similar pathways were affected, with an enrichment of cellular movement and an abrogation of the basal-like/squamous gene expression profile,^30^ as indicated by IPA and GSEA (Figures 4A–4D). These findings are confirmatory of the observations made in *SERPINB3*-transgene-expressing PDAC cells, as well as *SERPINB3*-high and basal-like/squamous PDAC tumors (Figure 2), by showing that SERPINB3 knockout and MYC signaling inhibition have the opposite effects. In a cell-culture-based assay, SERPINB3 knockout yielded a significant inhibition of the invasive ability of PDAC cell lines (Figure 4E).

Previous reports proposed that SERPINB3 increases MYC signaling,^21,24,25^ yet the underlying mechanism remains elusive. MYC degradation is predominantly governed by two established pathways: calpain-dependent cleavage and proteasomal degradation.^32^ Given that SERPINB3 functions as a serine/cysteine protease inhibitor,^18^ and calpain is a cysteine protease,^33^ we explored whether SERPINB3 inhibits calpain, thereby curbing MYC protein degradation (Figure 4F). To pursue this hypothesis, we examined the impact of SERPINB3 on calpain activity. Evidently, SERPINB3 overexpression diminished calpain activity, while its knockout heightened the activity in PDAC cells (Figure 4G). These findings collectively suggest that SERPINB3 contributes to the maintenance of basal-like/squamous differentiation in PDAC by stabilizing the MYC protein through calpain inhibition.

### Upregulation of acylcarnitine/carnitine and amino acid metabolism in SERPINB3-high and basal-like/squamous PDAC

**Figure 5.**
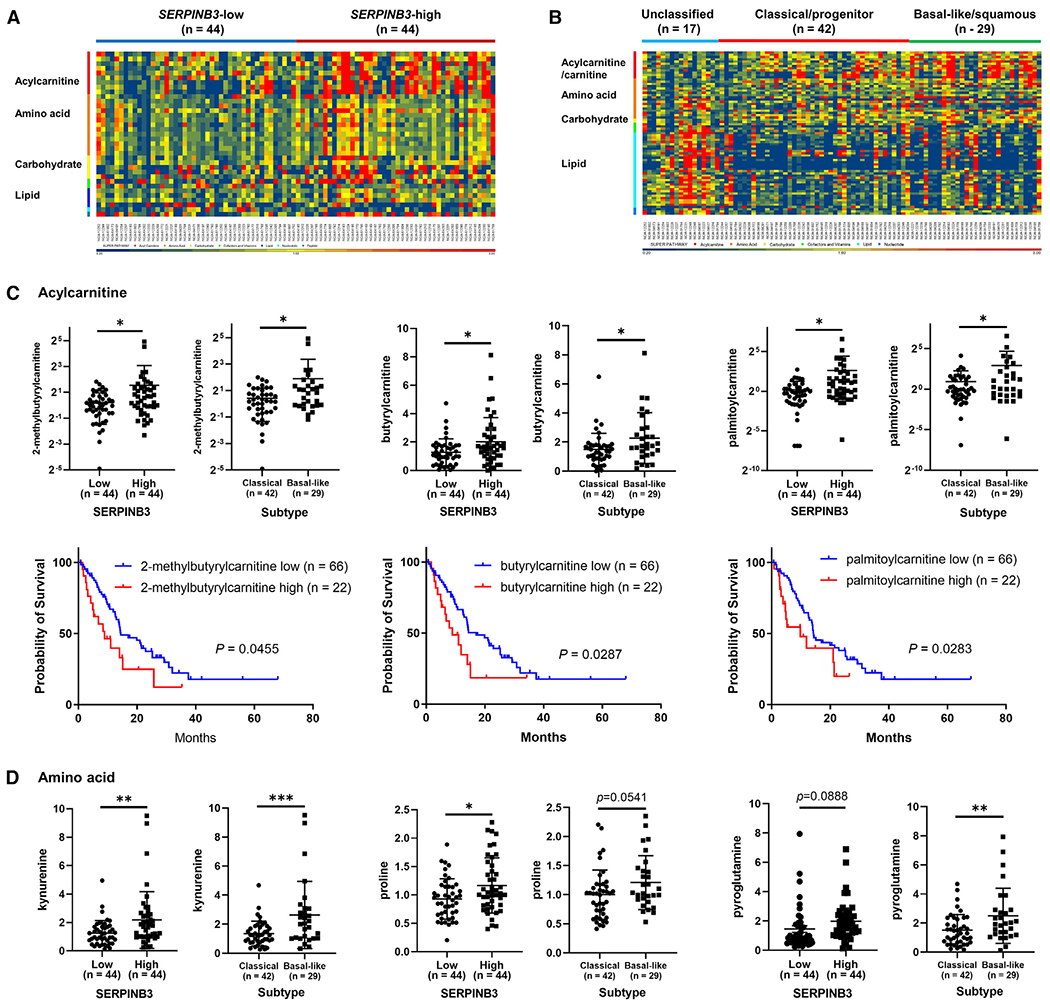
Upregulation of acylcarnitine/carnitine and amino acid metabolism in both SERPINB3-high and basal-like/squamous PDAC tumors (A and B) Heatmaps show metabolite patterns in human PDAC (n = 88) with high and low *SERPINB3* expression level (A) or by tumor subtype (B). Red shows upregulation of metabolites, blue indicates low abundance. In (A), 32 metabolites were significantly increased and 3 were decreased in *SERPINB3*-high tumors compared with *SERPINB3*-low tumors (p < 0.05). *SERPINB3*-low, n = 44; *SERPINB3*-high, n = 44. In (B), 15 metabolites were significantly increased and 2 were decreased in the basal-like/squamous subtype compared with the classical/progenitor subtype (p < 0.05). Unclassified subtype, n = 17; classical/progenitor subtype, n = 42; basal-like/squamous subtype, n = 29. Acylcarnitines/carnitine, amino acids, and carbohydrates are increased in *SERPINB3*-high PDAC tumors and also in basal-like/squamous PDAC tumors. (C and D) Abundance patterns for individual acylcarnitines (C) and amino acids (D) by tumor SERPINB3 status and subtype. Data are presented as the mean ± SD. In the graphs of 2-methylbutyrylcarnitine and palmitoylcarnitine, negative values of standard deviation (−SD) are not shown on the logarithmic y axis; *p < 0.05, **p < 0.01, ***p < 0.005 by unpaired two-tailed Student’s t test. The bottom row in (C) shows Kaplan-Meier plots and log-rank test results demonstrating the association of each acylcarnitine with PDAC patient survival. The comparison was conducted between patients in the upper 25% quartile and the lower 75% of each acylcarnitine level. Higher levels of 2-methylbutyrylcarnitine, butyrylcarnitine, and palmitoylcarnitine were associated with poor patient survival. See also Tables S3 and S4.

We next explored how SERPINB3 may influence the metabolism of PDAC by metabolic profiling of tumors from 88 patients in our NCI-UMD-German cohort (Table S3). Thirty-two metabolites were significantly increased and 3 were decreased in *SERPINB3*-high tumors, compared with *SERPINB3*-low tumors (Figure 5A; Table S4). Furthermore, 15 metabolites were significantly increased and 2 were decreased in the basal-like/squamous subtype compared with the classical/progenitor subtype (Figure 5B; Table S4). Among the 32 upregulated metabolites in *SERPINB3*-high tumors and the 15 upregulated metabolites in tumors of the basal-like/squamous subtype, 11 metabolites were common in both groups and included carnitine (a branched non-essential amino acid)/acylcarnitines (carnitine derivatives), amino acids, and carbohydrates (Figures 5A–5D). Moreover, the upregulation of several acylcarnitines was further associated with decreased patient survival (Figure 5C). In contrast, the unclassified subtype displayed significant upregulation of metabolites related to lipid metabolism (Figure 5B; Table S4), which were distinct from the significantly different metabolites in *SERPINB3*-high tumors.

**Figure 6.**
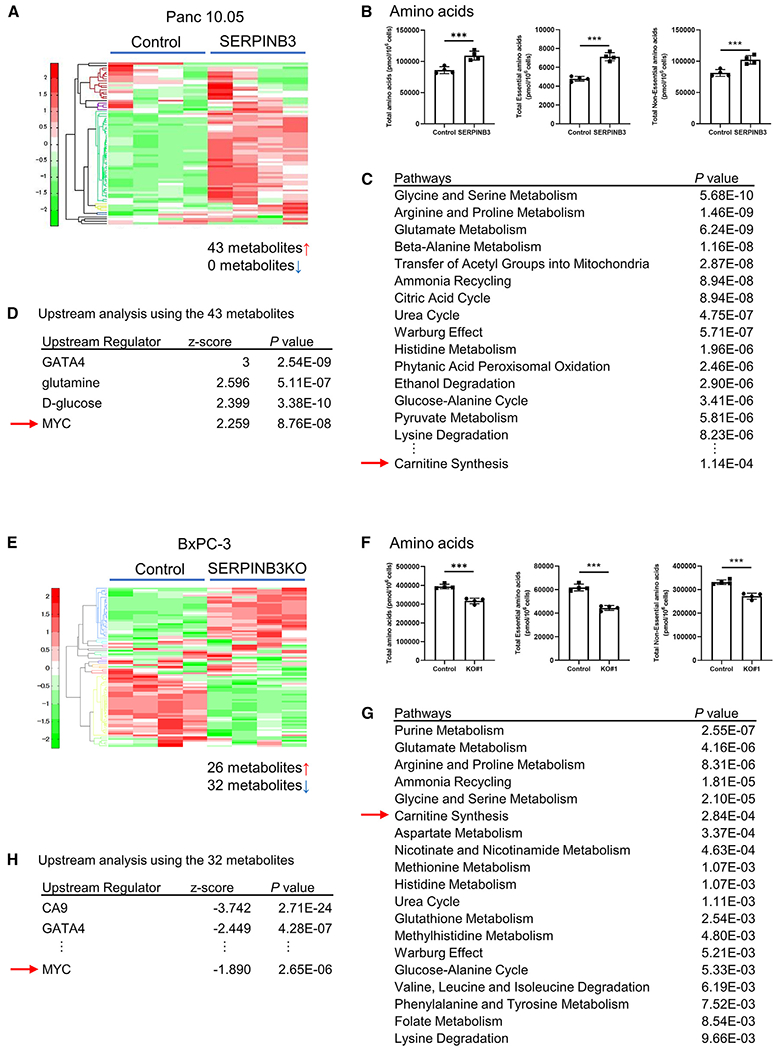
SERPINB3 promotes carnitine and amino acid metabolism in PDAC cells (A and B) Metabolome analysis covering 116 cancer-related metabolites in SERPINB3-overexpressing Panc 10.05 cells. In SERPINB3-overexpressing Panc 10.05 cells, 43 metabolites were significantly elevated compared with control cells (p < 0.05). The graphs highlight the increase in amino acid metabolism. Data are presented as the mean ± SD (n = 4 for each group); ***p < 0.005 by unpaired two-tailed Student’s t test. (C) Pathway enrichment scores using MetaboAnalyst 5.0 with 43 significantly elevated metabolites as input. It is shown that amino acid and carnitine metabolism and the Warburg effect are upregulated in SERPINB3-overexpressing Panc 10.05 cells. (D) Prediction of upstream regulators of metabolism in SERPINB3-overexpressing Panc 10.05 cells. The upstream analysis by IPA using the 43 input metabolites predicts upregulated MYC signaling in these cells. (E and F) Metabolome analysis covering 116 cancer-related metabolites in SERPINB3-knockout BxPC-3 cells. In these cells, 32 metabolites were significantly downregulated compared with control cells (p < 0.05). The graphs highlight the decrease in amino acid metabolism. Data are presented as the mean ± SD (n = 4 for each group); ***p < 0.005 by unpaired two-tailed Student’s t test. (G) Pathway enrichment scores using MetaboAnalyst 5.0 with 32 significantly decreased metabolites as input. It is shown that amino acid and carnitine metabolism and the Warburg effect are downregulated in SERPINB3-knockout BxPC-3 cells. (H) Prediction of upstream regulators of metabolism in SERPINB3-knockout BxPC-3 cells. The upstream analysis by IPA using the 32 input metabolites predicts downregulated MYC signaling in these cells. IPA, Ingenuity pathway analysis. See also Figures S4 and S5; Tables S5 and S6.

To continue our investigations into the role of the metabolites that are involved in the SERPINB3 and basal-like/squamous subtype, we performed a global metabolome analysis of 116 cancer-related metabolites with absolute quantitation. In SERPINB3-overexpressing Panc 10.05 cells, 43 metabolites were increased compared with control cells (Figure 6A; Table S5), notably encompassing amino acids (Figure 6B). Pathway enrichment analysis using MetaboAnalyst 5.0 (https://www.metaboanalyst.ca) with 43 input metabolites indicated the upregulation of carnitine and amino acid metabolism and the Warburg effect in SERPINB3-overexpressing Panc 10.05 cells (Figure 6C; Table S5). Upstream regulator analysis utilizing the 43 metabolites as input for IPA predicted the activation of MYC signaling by SERPINB3 as a mechanism underlying these changes in metabolism (Figure 6D; Table S6), further corroborating our observations. Notably, MYC signaling emerged as the sole regulator of SERPINB3 involved in basal-like/squamous PDAC differentiation (Figure 2) and the associated metabolic alterations (Figure 6). These findings were largely replicated in SERPINB3-overexpressing AsPC-1 and MIAPaCa-2 cells (Figure S4; Tables S5 and S6). The findings from our gain-of-function experiments were validated using SERPINB3-knockout cell lines, showing opposite effects of SERPINB3 knockout on metabolism compared with gain of function (Figures 6E–6H and S5; Tables S5 and S6). Collectively, these results underscore the role of the SERPINB3-MYC axis in sustaining the metabolic characteristics of basal-like/squamous PDAC.

### SERPINB3-associated metabolism enhances disease progression in PDAC

**Figure 7.**
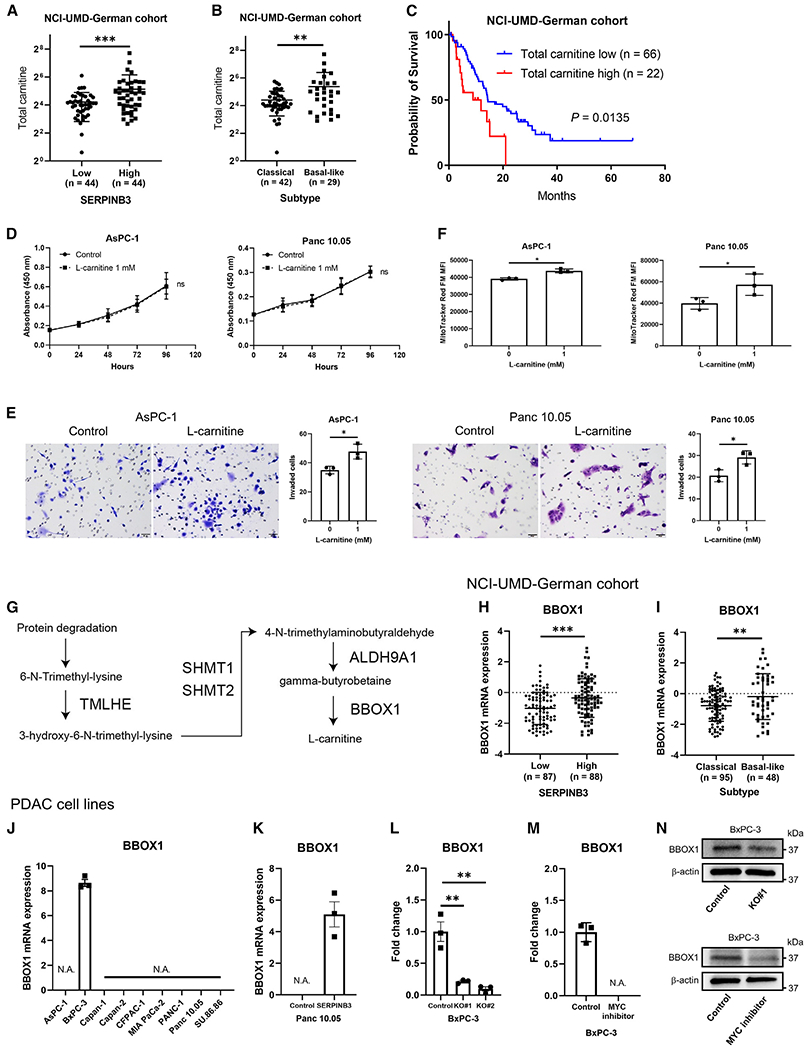
Carnitine enhances the disease progression in PDAC (A and B) Total carnitine is elevated in both *SERPINB3*-high and basal-like/squamous PDAC in the NCI-UMD-German cohort. Data are presented as the mean ± SD (*SERPINB3*-low, n = 44; *SERPINB3*-high, n = 44; classical/progenitor subtype, n = 42; basal-like/squamous subtype, n = 29). Negative values of standard deviation (−SD) are not shown on the logarithmic y axis; **p < 0.01, ***p < 0.005 by unpaired two-tailed Student’s t test. (C) Kaplan-Meier plot and log-rank test showing that high total carnitine associates with poor survival of PDAC patients (the upper 25% quartile versus the lower 75%; total carnitine low <30.8, high ≥30.8). (D) L-carnitine has no effect on proliferation of PDAC cell lines (CCK-8/WST-8 assay). Data are presented as the mean ± SD of three independent experiments; ns, not significant, by two-way ANOVA. (E) L-carnitine increases invasiveness of PDAC cell lines. Cells that passed through a cell culture insert membrane coated with Matrigel were fixed, and the cells were counted. Data are presented as the mean ± SD of three independent experiments; *p < 0.05 by unpaired two-tailed Student’s t test. (F) Increased mitochondrial membrane potential in PDAC cell lines cultured with 1 mM L-carnitine for 48 h. Mitochondrial membrane potential was quantified in cells using MitoTracker Red FM and flow cytometry. Data are presented as the mean ± SD (n = 3 for each group); *p < 0.05 by unpaired two-tailed Student’s t test. (G) *TMLHE/TMLD, HTMLA* (*SHMT1* and *SHMT2*), *ALDHA9A1/TMABADH*, and *BBOX1/BBH* are involved in L-carnitine synthesis.^36^ (H and I) Expression of *BBOX1* in clinical PDAC samples from the NCI-UMD-German cohort. *BBOX1* was upregulated in *SERPINB3*-high PDAC and the basal-like/squamous subtype. Data are presented as the mean ± SD; **p < 0.01, ***p < 0.005 by unpaired two-tailed Student’s t test. (J–M) Quantification of *BBOX1* expression in human PDAC cell lines using qPCR. BxPC-3 reigns as the foremost representative of the basal-like PDAC subtype^31^ and was the sole cell line expressing endogenous *BBOX1* (J). *BBOX1* was upregulated in SERPINB3-overexpressing Panc 10.05 cells (K) and downregulated in SERPINB3-knockout BxPC-3 cells (L). The supplementation of a MYC inhibitor (10058-F4, 100 μM) in the medium downregulated *BBOX1* expression at the mRNA level in BxPC-3 (M). Data are presented as the mean ± SD of three independent experiments; **p < 0.01 by unpaired one-way ANOVA. N.A. (not applicable), below the detection limit. (N) BBOX1 protein was downregulated in BxPC-3 through SERPINB3 knockout or the addition of a MYC inhibitor (10058-F4, 100 μM) to the medium. Total carnitine is the sum of all acylcarnitines/carnitine in the metabolome dataset: 2-methylbutyrylcarnitine (C5), acetylcarnitine, butyrylcarnitine, carnitine, decanoylcarnitine, deoxycarnitine, hexanoylcarnitine, isobutyrylcarnitine, isovalerylcarnitine, octanoylcarnitine, oleoylcarnitine, palmitoylcarnitine, propionylcarnitine, stearoylcarnitine, succinylcarnitine, and valerylcarnitine. See also Figures S6 and S7.

Based on the metabolomic profiles of patient tumors and PDAC cells, we focused on carnitine/acylcarnitines and amino acids for further functional investigations. We hypothesized that the sum of carnitine and acylcarnitines in the dataset (total carnitine) may reflect the function of carnitine/acylcarnitines more comprehensively than each carnitine or acylcarnitine alone. With this approach, we found that total carnitine was significantly increased in *SERPINB3*-high tumors and the basal-like/squamous subtype (Figures 7A and 7B). Moreover, higher levels of total carnitine were associated with decreased patient survival (Figure 7C). Exposing PDAC cells to L-carnitine showed that it did not have any effect on proliferation; however, L-carnitine resulted in enhanced invasiveness (Figures 7D and 7E), further supporting our observation that SERPINB3 may enhance the metastatic process more so than the growth of primary PDAC tumors. L-carnitine treatment of PDAC cells resulted in increased mitochondrial membrane potential (Figure 7F), which is consistent with previous observations.^34^ This effect could potentially be attributed to L-carnitine’s role as a redox system and radical scavenger^35^ in countering the heightened levels of ROS in SERPINB3-expressing PDAC cells, as illustrated in Figure S2. Accordingly, SERPINB3-overexpressing PDAC cells also exhibited an elevated mitochondrial membrane potential (Figure S6A). Previous studies showed that carnitine exerts a protective role in cells by averting the buildup of fatty acyl-CoA, a potential source of free radicals and cellular toxicity.^35^ This protection is achieved through the conversion of fatty acyl-CoA into acylcarnitines.^35^ To explore the potential of L-carnitine in functioning as a redox system to counteract ROS, we conducted an experiment involving the supplementation of the medium with palmitic acid and L-carnitine. The exposure to palmitic acid led to elevated mitochondrial ROS levels, which were subsequently mitigated by the presence of L-carnitine (Figure S6B). Moreover, proline/hydroxyproline was distinctively increased in *SERPINB3*-high PDAC tumors, the basal-like/squamous subtype, and SERPINB3-overexpressing PDAC cells (Figures 5D and S6C); therefore, we examined if hydroxyproline may alter the phenotype of PDAC cells. Hydroxyproline did not show any effect on proliferation of PDAC cell lines; however, it increased invasiveness (Figures S6D and S6E).

Having made these observations, we continued to study why carnitine/acylcarnitines might be upregulated in basal-like/squamous PDAC and conducted a comprehensive analysis of genes implicated in L-carnitine synthesis,^36^ specifically *TMLHE, SHMT1, SHMT2, ALDH9A1*, and *BBOX1* (Figure 7G), within both our NCI-UMD-German cohort and the PDAC cell lines. Among them (Figures 7 and S7), *BBOX1* was consistently upregulated in *SERPINB3*-high PDAC (Figure 7H) and the basal-like/squamous subtype (Figure 7I) in our NCI-UMD-German cohort. Among the PDAC cell lines that we investigated, BxPC-3, which exhibited the highest endogenous SERPINB3 expression (Figure S1A) and is the foremost representative of the basal-like/squamous PDAC subtype,^31^ was the only cell line with endogenous *BBOX1* expression (Figure 7J). An induction of *BBOX1* via SERPINB3 upregulation was observed in the Panc 10.05 cells (Figure 7K). In contrast, BBOX1 expression was downregulated in BxPC-3 through SERPINB3 knockout or treatment with a MYC inhibitor (10058-F4) at both mRNA and protein levels (Figures 7L–7N), confirming *BBOX1* as a target gene of MYC. Our findings indicate that the elevated carnitine synthesis in the presence of SERPINB3 may occur because of Myc-induced BBOX1 upregulation and could be further enhanced by an augmented amino acid synthesis, because L-carnitine is derived from lysine and methionine.

## DISCUSSION

Recently, a comprehensive investigation of PDAC proposed its classification into two molecular subtypes: classical/progenitor and basal-like/squamous.^6^ However, neither this study nor others fully elucidated the key genes promoting subtype differentiation or the metabolism associated with these subtypes. In the present study, we report that SERPINB3 is upregulated in the basal-like/squamous PDAC subtype and associated with decreased patient survival. In a PDAC preclinical model, SERPINB3 increased metastasis. Molecular analysis of primary tumor xenografts showed upregulation of pathways related to angiogenesis, oxidative stress, and metastasis. Metabolome analyses identified a specific metabolic signature closely tied to both SERPINB3 and the basal-like/squamous subtype, including an upregulation of carnitine and amino acid metabolism and the Warburg effect. Further analysis revealed that SERPINB3 induces the basal-like/squamous subtype and associated metabolic reprogramming through MYC activation. Taken together, these findings suggest that SERPINB3 contributes to the maintenance of the basal-like/squamous PDAC subtype.

Serine protease inhibitors (serpins) constitute one of the largest superfamilies of protease inhibitors, playing critical roles in the regulation of key pathways, including coagulation and inflammation. Although the majority of serpins function as serine protease inhibitors, some also inhibit caspases^37^ and papain-like cysteine proteases, including cathepsin L, S, and K, and papain.^38,39^ SERPINB3, also known as SCCA1, was initially described as a tumor-specific antigen in squamous cell carcinoma of the uterine cervix.^40^ SERPINB3 was later found to be upregulated in squamous cell carcinoma of various organs,^18,39^ as well as in other cancers, including hepatocellular carcinoma,^25^ cholangiocarcinoma,^23^ PDAC,^27^ glioblastoma,^24^ and breast cancer.^26^ In cancer, SERPINB3 has various functions, including inhibition of radiation- or anti-cancer-drug-induced apoptosis, protein degradation, and intratumor infiltration of natural killer cells^18,25,39^ and promotion of metabolic reprogramming, angiogenesis, epithelial-mesenchymal transition, and secretion of inflammatory mediators.^19,23–27^ SERPINB3 also upregulates MYC,^21,22^ thereby contributing to the maintenance of cancer stemness.^23,24^ In line with these discoveries, our study revealed that SERPINB3 modulates metabolism and maintains the basal-like/squamous PDAC subtype by activating the MYC signaling pathway through calpain inhibition. In the orthotopic mouse model of PDAC, SEPRINB3 led to the activation of stroma with upregulation of angiogenesis and tumor microenvironmental pathways. Thus, SERPINB3 may play a role not only in the development and maintenance of the basal-like/squamous subtype in PDAC but also in inducing an “activated” stroma, as described previously.^4^

Cancer cells increase aerobic glycolysis, amino acid and lipid synthesis, and macromolecule synthesis using the upregulation of the pentose phosphate pathway.^10,11^ Genetic alterations are one of the drivers of such metabolic adaptations and may include amplification of the *MYC* locus. These adaptations result in appropriate maintenance of redox homeostasis, ATP generation, and promotion of their survival. MYC plays a central role in the activation of glycolysis and amino acid metabolism.^11^ MYC activates the transcription of glycolytic genes. MYC also increases the availability of essential amino acids through induction of amino acid transporters, such as SLC1A5, SLC7A5, and SLC43A1. The upregulation of tryptophan uptake leads to an increase in the activity of the kynurenine pathway. For the metabolism of non-essential amino acids, including glutamine, proline, serine, and glycine, MYC promotes biosynthesis/transport by upregulating the synthetic enzymes/transporters. Our findings revealed that heightened SERPINB3 expression in PDAC leads to a marked upregulation in the metabolism of both essential and non-essential amino acids, as well as the metabolites associated with the Warburg effect. Consistently, similar association of metabolic adaptation was found in patient tumors of the basal-like/squamous subtype. Conversely, the knockout of SERPINB3 resulted in a downregulation of these metabolites.

Furthermore, upstream regulator analysis predicted MYC signaling as a potential mechanism leading to these metabolic alterations. MYC may upregulate carnitine metabolism.^11,41–43^ Notably, MYC was found to upregulate carnitine uptake in triple-negative breast cancer,^41^ a representative type of basal-like breast cancer. In agreement with this finding and the candidate role of MYC as a regulator, downstream of SERPINB3, we observed the coherent upregulation of carnitine/acylcarnitine metabolism in the basal-like/squamous subtype of PDAC or when SERPINB3 was upregulated. Carnitine metabolism was downregulated in SERPINB3-knockout PDAC cells. Thus, upregulation of the carnitine/acylcarnitine metabolism pathway might be one of the features of basal-like subtype tumors across the cancer spectrum.

Carnitine, a branched non-essential amino acid, is derived from two amino acids, lysine and methionine.^43–45^ We showed that *BBOX1*, a carnitine-synthesis gene, is the target of the SERPINB3-MYC axis in PDAC. Intriguingly, BBOX1’s upregulation is not limited to PDAC but extends to triple-negative breast cancer,^46,47^ a disease that is also known to be intricately linked to MYC activation. This convergence underscores the broader implications of this pathway. The increase in carnitine/acylcarnitine levels observed in SERPINB3-high PDAC and the basal-like/squamous subtype may stem from the concurrent elevation in amino acid metabolism and the heightened expression of BBOX1. L-carnitine plays a crucial role in facilitating the import of fatty acids into mitochondria, enabling subsequent fatty acid oxidation.^43–45^ Previous studies reported a protective function of L-carnitine within cells, effectively preventing the accumulation of acyl-CoA, a by-product of fatty acids that could potentially generate free radicals and cellular toxicity.^35^ This safeguarding is accomplished by converting acyl-CoA into acylcarnitine derivatives.^35^ As a result, the intrinsic pool of carnitine encompasses not only L-carnitine but also an array of diverse acylcarnitines. Several studies showed that carnitine increases mitochondrial membrane potential^34^ and resistance against oxidative stress.^48,49^ In addition, carnitine serves as a mediator for expelling surplus carbon residues—specifically acetyl-CoA—by transforming them into acylcarnitines, a process that facilitates both glucose utilization and metabolic adaptability.^44,50^ This versatility extends further to the metabolism of branched-chain amino acids, showcasing the multifaceted involvement of acylcarnitines in cellular processes.^45^ Our study unveiled that SERPINB3-high PDAC and the basal-like/squamous subtype do not exhibit the upregulation of lipogenesis/fatty acid oxidation. Instead, carnitine emerges as a pivotal redox system against fatty acids. Although the upregulation of carnitine/acylcarnitine metabolism in cancer has previously been documented, together with a role for carnitine/acylcarnitine metabolism in cancer progression,^43–45,49,51^ to our knowledge our study is the first to show its potential role in PDAC.

This study has the potential to advance the diagnostic process for the molecular subtype of PDAC by identifying a key driver of basal-like/squamous PDAC. While molecular subtyping has been extensively studied and proven clinically valuable in breast cancer,^52^ the situation is different for PDAC. Breast cancer is generally classified into four distinct molecular subtypes by testing the expression of several proteins by IHC, which can help guide appropriate therapeutic interventions. In contrast, molecular subtyping is still in the research phase in PDAC, primarily relying on whole-exome sequencing. However, our study revealed that SERPINB3 could induce both gene and metabolic profiles of the basal-like/squamous subtype. By testing the expression of several genes or proteins, including SERPINB3, through techniques such as IHC or qPCR, it may be possible to diagnose molecular subtypes of PDAC in a cost-effective manner suitable for clinical applications.

In summary, we provide evidence that SERPINB3-MYC signaling promotes basal-like/squamous differentiation and associated metabolic reprogramming. The observations argue that SERPINB3 is a candidate diagnostic and therapeutic target for PDAC.

### Limitations of the study

A limitation of our study pertains to the difficulty of showing the correlation between SERPINB3 expression and metastatic PDAC in the patient cohorts. This discrepancy with our animal model findings arises from diverse factors, including the differing temporal dynamics of tumor development, detection, and diagnosis in controlled mouse models and complex clinical scenarios. Additional limitations arise from the observational nature of our patient data, although our experiments have provided valuable insights into the SERPINB3-MYC axis. It is important to note that our findings might not fully capture the complexity of interactions within the context of PDAC patients, as the tumor microenvironment may influence PDAC metabolism.

## STAR★METHODS

### RESOURCE AVAILABILITY

#### Lead contact

Further information and requests for resources and reagents should be directed to and will be fulfilled by the lead contact, Yuuki Ohara (yuuki.oohara.1196@gmail.com).

#### Materials availability

All plasmids generated in this study are available from the lead contact with a completed transfer agreement.

#### Data and code availability

- The raw RNA sequencing data are publicly available at the NCBI’s Gene Expression Omnibus (GEO) database under accession number (GSE223909) [https://www.ncbi.nlm.nih.gov/geo/query/acc.cgi?acc=GSE223909]. This SuperSeries is composed of the following SubSeries GSE224564; GSE223908; GSE223536. The raw metabolome data in PDAC cells have been deposited at OSF and are publicly available at [https://osf.io/3jyut/?view_only=fed315a5a79f4cc283790eefc73fca3f].
- This paper does not report original code.
- Any additional information required to reanalyze the data reported in this paper is available from the lead contact upon request.

### EXPERIMENTAL MODEL AND STUDY PARTICIPANT DETAILS

#### Cell lines and culture condition

We purchased human PDAC cell lines from American Type Culture Collection (ATCC), Rockville, Maryland. Cell lines were authenticated by short tandem repeat (STR) analysis at ATCC, per request, and were mycoplasma-free. CFPAC-1 and Capan-1 cell lines were grown in IMDM with 10% FBS, and 1% penicillin–streptomycin. Capan-2 cell line was grown in McCoy’s 5A (Modified) medium with 10% FBS, and 1% penicillin–streptomycin. The remaining PDAC cell lines were cultured in RPMI 1640 medium with GlutaMax^™^, 10% FBS, and 1% penicillin–streptomycin in a humidified incubator containing 5% CO_2_ at 37 °C. All reagents for cell culture were purchased from Thermo Fisher Scientific (Waltham, MA).

#### Orthotopic injection of AsPC-1 cells into the pancreas of mice for tumor growth

Animal experiments and maintenance conformed to the guidelines of the Animal Care and Use Committee at NCI and the American Association of Laboratory Animal Care. Each experiment was approved by ACUC at NCI, Frederick, MD. NOD-SCID mice were purchased from Jackson Laboratory (Bar Harbor, ME). 5×10^5^ of either SERPINB3-overexpressing or control AsPC-1 cells were orthotopically transplanted in the pancreas of NOD-SCID mice (male, n = 5; female, n = 5 in each group). The mice were euthanized at 5 weeks after transplantation. A complete necropsy was performed, and the primary tumors were harvested and weighed. Paraffin-embedded sections of the pancreas, liver, and lungs were prepared and evaluated for any pathological changes including metastasis.

### METHOD DETAILS

#### Human samples

Pancreatic tumor tissues from resected PDAC patients were collected at the University of Maryland Medical System (UMMS) in Baltimore, MD, through an NCI-UMD resource contract and at the University Medical Center Göttingen, Germany. PDAC histopathology was determined by board-certified pathologists. The use of these clinical samples has been approved by the NCI-Office of the Human Subject Research Protection (OHSRP, Exempt#4678) at the NIH (Bethesda, MD).

#### Public datasets

Transcriptome datasets derived from the “Bailey” cohort (GSE36924)^3^, the “Moffitt” cohort (GSE71729)^4^, and our NCI-UMD-German cohort (GSE183795)^17^ were used for identifying genes of origin for the basal-like/squamous subtype. We used the Partek Genomics Suite 7.0 (Partek Inc., Chesterfield, MO) for comparing the basal-like/squamous subtype with the classical/progenitor subtype and performing COX regression for survival analysis.

### Gene expression analysis

RNA sequencing data from patient tumors, PDAC cell lines, and tumor xenografts: RNA-seq were performed and the RNA-seq data were respectively deposited in the NCBI’s GEO database under accession number GSE224564, GSE223908, and GSE223536. Ingenuity pathway analysis (IPA, QIAGEN, Venlo, Netherlands) and Gene Set Enrichment Analysis (GSEA) were used for the enrichment analysis.

1. RNA sequencing data from patient tumors: For bulk RNA sequencing on human PDAC patient samples, libraries were prepared by the Sequencing Facility at NCI-Leidos using the TruSeq Stranded mRNA Kit (Illumina, San Diego, CA) and sequenced paired-end on NovaSeq (Illumina) with 2 x 150 bp read lengths. About 51 to 122 million paired-end reads in total were generated with a base call quality of Q30 and above. The sequence reads in FASTQ format were aligned to the human reference genome hg38 using STAR and RSEM to obtain gene expression as transcript per million and FPKM mapped reads. Differential expression was performed using DESeq2.The RNA-seq data from patient tumors were deposited in the NCBI’s GEO database under accession number GSE224564.
2. RNA sequencing data from PDAC cell lines: We performed quadruplicate RNA-sequencing on human PDAC cell lines (AsPC-1 +/− SERPINB3; Panc 10.05 +/− SERPINB3; BxPC-3 +/− SERPINB3 knockout; AsPC-1 SERPINB3 +/− a MYC inhibitor 100 μM; Panc 10.05 SERPINB3 +/− a MYC inhibitor 100 μM). The MYC inhibitor 10058-F4 was purchased from MedChemExpress LLC (Monmouth Junction, NJ, HY-12702), and its corresponding solvent, dimethyl sulfoxide (DMSO), was used as the control. Cells were cultured with regular media for 72 hours before RNA extraction. For AsPC-1 +/− SERPINB3 and Panc 10.05 +/− SERPINB3, libraries were prepared using the TruSeq Stranded mRNA Kit (Illumina) and sequenced paired-end on NextSeq (Illumina) with 2 x 150 bp read lengths. About 22 to 29 million paired-end reads were generated with a base call quality of Q30 and above. For BxPC-3 +/− SERPINB3 knockout, AsPC-1 SERPINB3 +/− a MYC inhibitor, and Panc 10.05 SERPINB3 +/− a MYC inhibitor, libraries were prepared using the TruSeq Stranded mRNA Kit (Illumina) and sequenced paired-end on NovaSeq 6000 S2 (Illumina) with 2 x 150 bp read lengths. About 86 to 130 million paired-end reads were generated with a base call quality of Q30 and above. Data analysis was processed, as described in human PDAC patient samples. The RNA-seq data for human PDAC cell lines were deposited in the NCBI’s GEO database under accession number GSE223908.
3. RNA sequencing data from tumor xenografts: RNA sequencing on the pancreatic xenografts in the orthotopic mouse model using PDAC cells (AsPC-1 control; n = 6, AsPC-1 SERPINB3; n = 6) was also performed following isolation of RNA with the RNeasy Plus Mini Kit (QIAGEN, Venlo, Netherlands). Sequencing was performed on NovaSeq 6000 SP (Illumina) with 2 x 150 bp read lengths. About 133 to 164 million paired-end reads in total were generated with a base call quality of Q30 and above. The sequence reads in FASTQ format were aligned to the human reference genome hg38 and the mouse reference genome mm10 using STAR and RSEM to obtain gene expression as transcript per million and FPKM mapped reads. Differential expression was performed using DESeq2. The RNA-seq data for the xenografts were deposited in the NCBI’s GEO database under accession number GSE223536.

#### PDAC subtype assignment using the published “Moffitt” gene set

A list of signature genes was obtained as earlier described by *Moffitt* et al.^4^ We obtained the expression pattern of the signature genes from RNA sequencing and assigned those to each tumor as a score and performed hierarchical clustering with Euclidean distance and the ward D2 clustering algorithm. The approach split the tumors into 3 main subtypes that we manually curated as classical/progenitor, basal-like/squamous, and unclassified.

#### Metabolic profiling and data analysis of tumor samples from PDAC patients

The metabolic profiling of our tumor samples from PDAC patients was conducted by Metabolon Inc. (Morrisville, NC) using a standard protocol.^55–58^ For the current study, three existing metabolome profiling datasets (n = 33, n = 31, and n = 33) were combined and 235 common metabolites of known identity were analyzed. The merged data was normalized by assigning the median of each compound level to equal to one for each dataset termed “block normalization”. We imputed missing data with the minimum observed value for each metabolite in each dataset and the combined dataset was log2-transformed. Only patient samples with RNA sequencing and follow up data were included in this study (n = 88). The dataset served for further analysis (Table S3).

#### Metabolome analysis of PDAC cell lines

Absolute concentration of 116 metabolites was examined in various cell lines using the metabolome analysis package “Carcinoscope” provided by Human Metabolome Technologies, Inc. (HMT) (Boston, MA). For metabolite extraction, cell extracts were obtained using the standard manufacturer’s protocol.^59^ PDAC cells were seeded on a 100 mm dish and incubated in a specific medium (IMDM for CFPAC-1 and RPMI 1640 medium with GlutaMax^™^ for the remaining cell lines), supplemented with 10% FBS and 1% penicillin–streptomycin for 72 hours in an incubator. The initial cell seeding numbers were as follows: AsPC-1 +/− SERPINB3 1 x 10^6^ cells; Panc 10.05 +/− SERPINB3 1 x 10^6^ cells; BxPC-3 +/− SERPINB3 knockout 1.5 x 10^6^ cells; CFPAC-1 +/− SERPINB3 knockout 1.5 x 10^6^ cells; Panc 10.05 +/− SERPINB3 knockout 1.5 x 10^6^ cells. After the removal of medium from a dish, 800 μl of methanol was added and incubated at room temperature for 30 sec. 550 μl of Internal Standard Solution were added and incubated at room temperature for 30 sec. One ml of the extracted solution was transferred to a 1.5 ml microtube and centrifuged at 4°C for 5 min (2300 x g). 350 μl of the supernatant was transferred into a centrifugal filter unit and centrifuged at 4°C for 5 hours (9100 x g). Filtered samples were stored at −80°C until shipping. Metabolome analysis was performed by HMT using capillary electrophoresis mass spectrometry (CE-MS). IPA and MetaboAnalyst 5.0 (https://www.metaboanalyst.ca)^53^ were used for the enrichment analysis.

#### Quantitative real-time PCR

The High-Capacity cDNA Reverse Transcription Kit (Thermo Fisher Scientific, Waltham, MA) was used to create first-strand cDNA from total RNA. Quantitative real-time PCR (qPCR) assays were performed on the CFX384 Touch Real-Time PCR Detection System (Bio-Rad Laboratories, Inc. Hercules, CA) using Taqman probes (Thermo Fisher Scientific): *SERPINB3* (Hs00199468_m1), *TMLHE* (Hs00379459_m1), *SHMT1* (Hs00541043_g1), *SHMT2* (Hs01059263_g1), *ALDH9A1* (Hs00997881_m1), *BBOX1* (Hs00187779_m1), and *GAPDH* (Hs99999905_m1).

#### SERPINB3 overexpression after lentiviral infection

The SERPINB3 construct (EX-F0390-Lv103) and the corresponding empty vector control (EX-NEG-Lv103) were purchased from Genecopoeia (Rockville, MD). To obtain stable cell lines overexpressing SERPINB3, PDAC cells (AsPC-1, MIA PaCa-2, and Panc 10.05) were infected with lentiviral particles produced by transfecting 293T cells using the lentiviral expression vectors and the Lenti-Pac^™^ HIV Expression Packaging system from Genecopoeia. The PDAC cells were selected with puromycin (4 μg/ml) to obtain stable clones.

#### Generation of SERPINB3-knockout PDAC cells

CRISPR/Cas9 constructs targeting SERPINB3 for knockout were generated by designing guide RNAs through sgRNA scorer 2.0.^60^ These guide RNAs were synthesized via *in vitro* transcription and then complexed with Cas9 protein. The activity of the guide RNAs was assessed in 293T cells using established methods,^61^ followed by high-throughput Illumina sequencing (Illumina MiSeq). A prominent candidate from this evaluation, the guide RNA sequence IVT-2407 (GAACAGGTCGAACATGAACTTGG), was selected for subsequent experiments. Oligonucleotides corresponding to IVT-2407 were annealed, phosphorylated, and ligated into pGMC00009 (Addgene, Watertown, MA, 195294) using T4 ligase. DH5α Competent Cells for Subcloning (Thermo Fisher Scientific) and the QIAfilter Plasmid Midi Kit (QIAGEN) were used for the cloning and purification of plasmids (plasmid names: pMC0235, non-targeting control; pMC0224, SERPINB3 knockout). Lentiviral particles were produced by transfecting 293 T cells using the plasmid and the Lenti-Pac^™^ HIV Expression Packaging system (Genecopoeia). Lentivirus was subsequently used to infect BxPC-3, CFPAC-1, and Panc 10.05 parent cell lines. Single-cell sorting of these cells was accomplished through the BD FACSAria IIu cell sorter (Becton, Dickinson and Company, San Jose, CA), followed by establishing single-cell clones that were puromycin-selected (4 μg/ml). Verification of SERPINB3 knockout was carried out using qPCR, western blot analysis, and genomic sequencing as elaborated below.

To assess editing in PDAC cell line clones, genomic DNA was extracted using DNeasy Blood & Tissue Kit (QIAGEN), and a 10 μL PCR reaction was performed using Primestar Max Polymerase (Takara Bio, Shiga, Japan, R045B) with 20 ng genomic DNA as template. The PCR product was purified using magnetic beads, and a barcoding PCR reaction was performed using the same polymerase. Sequencing was carried out on the Illumina MiSeq Nano kit (2 x 150 format). Data analysis was conducted using an in-house pipeline (https://github.com/rajchari2/ngs_amplicon_analysis)^61^, which was executed in the following sequence. Paired end reads were merged using FLASH^62^ and merged FASTQ files were mapped to the hg38 genome sequence. Sorted BAM files were analyzed for indels around the guide RNA target site and subsequently visualized using the Integrated Genomics Viewer (IGV).^54^ Further details on the used candidate guide RNA sequences and oligonucleotides are available in Table S7.

#### Cell proliferation assay

PDAC cells were seeded in a 96-well plate and the CCK-8/WST-8 assay was performed according to the manufacturer’s protocol (Dojindo Laboratories, Kumamoto, Japan), at 0, 24, 48, 72, and 96 hours after seeding. The absorbance was measured with SpectraMax^®^ ABS Plus microplate reader (Molecular Devices, San Jose, CA). Gemcitabine was purchased from MilliporeSigma (Burlington, MA, G6423).

#### Cell invasion assay

24-well Falcon^®^ Cell Culture Insert and Matrigel Matrix were purchased from Corning Inc. (Corning, NY). The membrane of the upper insert was coated by applying 100 μl of Matrigel matrix coating solution (Matrigel matrix: coating buffer = 1:39) for 2 hours according to the manufacturer’s protocol. PDAC cells (AsPC-1 10x10^4^ cells; BxPC-3 3x10^4^ cells; CFPAC-1 5x10^4^ cells; Panc 10.05 10x10^4^ cells) in 500 μl serum-free RPMI 1640 with GlutaMax^™^ were loaded into each upper insert, and 750 μl of RPMI 1640 with GlutaMax^™^ and 10% FBS were added to the lower chamber. We counted PDAC cells that passed through the cell culture insert membrane after 48 hours of incubation in a humidified incubator containing 5% CO2 at 37 °C. For fixation and staining of the cells, 100% methanol (Thermo Fisher Scientific) and Crystal violet solution (MilliporeSigma) were used. To examine the effect of L-carnitine and hydroxyproline, either 1 mM of L-carnitine or 0.5 mM of hydroxyproline (final conc.) was added into the lower chamber. L-carnitine (C0158) and hydroxyproline (H5534) were purchased from MilliporeSigma.

#### Measurement of mitochondria membrane potential and cytoplasmic ROS

PDAC cells were seeded in a 60 mm dish at 1x10^6^ cells with regular media and cultured for 48 hours. To examine the effects of L-carnitine, 1 mM L-carnitine (final conc.) was added to the culture media after PDAC cells were seeded. We used CellROX^™^ Deep Red Reagent (Thermo Fisher Scientific, C10422) for ROS measurement and MitoTracker^™^ Red FM (Thermo Fisher Scientific, M22425) for measurement of the mitochondrial membrane potential. In this process, PDAC cells were incubated with 5 μM of CellROX^™^ Deep Red or 100 nM of MitoTracker^™^ Red FM for 30 minutes at 37°C. After washing twice with PBS, we evaluated ROS or mitochondrial membrane potential using a SONY SA3800 flow cytometer (Sony Group Corporation, Tokyo, Japan). Data analysis was performed using FlowJo version 10.8.1 (Becton, Dickinson and Company, San Jose, NJ).

#### Measurement of mitochondrial ROS in PDAC cells

PDAC cells (AsPC-1 1x10^4^ cells; Panc 10.05 1x10^4^ cells) were seeded in a 96-well black plate (Thermo Fisher Scientific) containing regular growth media, with or without the addition of 1 mM L-carnitine, and incubated for 24 hours. Subsequently, palmitic acid was introduced into the wells and the cells were cultured for an additional 20 hours. Following this, the cells were exposed to 1 μM of MitoSOX^™^ Red (Thermo Fisher Scientific, M36008) for a duration of 10 minutes at 37°C. Fluorescence emission at 580 nm, with excitation at 510 nm, was measured using the EnSpire^™^ multimode plate reader (PerkinElmer, Waltham, MA).

For the preparation of conjugated palmitic acid-BSA complexes, we followed the established protocol,^63^ described in the following steps. A solution of 200 mM sodium palmitic acid in 95% ethanol was prepared by ensuring complete dissolution through vortexing. This solution was then diluted 25-fold in a 10% fatty acid-free BSA solution (MilliporeSigma), resulting in an 8 mM solution. The final molar ratio of palmitic acid to BSA was 5.3:1. Conjugation was carried out at a temperature of 40°C for a duration of 2 hours. The BSA-fatty acid conjugates were subsequently further diluted in cell culture media, resulting in a final palmitic acid concentration of 0.4 mM and 0.8 mM. Control BSA samples were generated by adding an equivalent volume of 95% ethanol to a 10% BSA solution. All prepared samples were aliquoted and promptly frozen at −20°C to maintain their integrity.

#### Calpain activity assay in PDAC cells

Calpain activity was assessed using the Calpain-Glo^™^ assay kit obtained from Promega (Madison, WI, G8502) in accordance with the manufacturer’s instructions and based on previous studies.^64,65^ PDAC cells (AsPC-1 1x10^4^ cells; BxPC-3 0.6x10^4^ cells; Panc 10.05 2x10^4^ cells) were seeded in a 96-well white plate (Thermo Fisher Scientific) with regular growth media and incubated for 24 hours. The Calpain-Glo reagent^™^ and Suc-LLVY-Glo^™^ substrate were mixed and incubated without Ca^2+^. Subsequently, the cell culture medium was aspirated, and 90 μl of the mixture was added to each well, followed by a 20-minute incubation at 37°C. For cell lysis, 10 μl of lysis buffer containing 9% Triton-X-100 and 1 mM of the calpain inhibitor Calpeptin (MedChemExpress LLC, Monmouth Junction, NJ) was introduced to the wells to prevent further substrate cleavage by calpain. Luminescence, indicative of cellular calpain activity, was measured using the EnSpire^™^ multimode plate reader (PerkinElmer, Waltham, MA).

#### Immunohistochemistry

Four μm thick paraffin-embedded sections were prepared for immunohistochemistry (IHC). Antigen retrieval was performed by heating the sections with a microwave in Target Retrieval Solution, pH 9 (Agilent Technologies, Santa Clara, CA). The sections were incubated with primary antibodies overnight at 4°C. The following antibodies were used: SERPINB3 (Abcam, Cambridge, United Kingdom, ab154971; 1:500), CD31 (Abcam, ab28364; 1:100), 8-hydroxy-2’-deoxyguanosine (8-OHdG) (Abcam, ab48508; 1:5000). Signals were amplified using the Dako EnVision+ System- HRP labeled polymer anti-mouse or rabbit antibody protocol. Color development was conducted with diaminobenzene (DAB, Agilent Technologies). For the human PDAC samples and the samples from the mouse PDAC xenograft model, the immunostaining of SERPINB3 was evaluated assigning the intensity and distribution scores.^66,67^ The intensity was assigned a score of 0–3, representing negative, weak, moderate, or strong expression and distribution was given a score of 0–4, with <10%, 10–30%, >30–50%, >50–80% or >80% cells showing SERPINB3 expression. Then the overall IHC score was obtained by multiplying the intensity and distribution scores. The density of microvessels in the orthotopic tumor xenografts was assessed using CD31 expression in endothelial cells. Oxidative stress was assessed by the percentage of 8-OHdG-positive tumor cells in the xenografts.

#### Immunoblotting

Cells were lysed with RIPA Lysis and Extraction Buffer (Thermo Fisher Scientific), supplemented with cOmplete^™^ Protease Inhibitor Cocktail (MilliporeSigma). Proteins were electrophoresed under reducing conditions on 4–15% polyacrylamide gels (Bio-Rad Laboratories, Inc., Hercules, CA) and then transferred onto a nitrocellulose membrane (Bio-Rad Laboratories, Inc.). This membrane was incubated for 60 min with SuperBlock^™^ Blocking Buffer (Thermo Fisher Scientific) at room temperature. Incubation with primary antibody was carried out overnight at 4°C. The following primary antibodies were used: SERPINB3 (R & D Systems, Minneapolis, MN, MAB6528; 1:500), SERPINB3 (Thermo Fisher Scientific, PA5-30164; 1:2000), c-Myc (MilliporeSigma, M4439, 1:2000), BBOX1 (MilliporeSigma, WH0008424M1, 1:200), Cas9 (Cell Signaling Technology, Inc., Beverly, MA, 14697T, 1:1000), and β-Actin (MilliporeSigma, A5441, 1:2000). The membrane was incubated with secondary ECL anti-rabbit or anti-mouse IgG HRP-linked antibody (GE Healthcare, Pittsburgh, PA) for 1 hour at room temperature. Protein was visualized using a SuperSignal^™^ West Dura Extended Duration Substrate (Thermo Fisher Scientific).

### QUANTIFICATION AND STATISTICAL ANALYSIS

#### Statistical analysis

We performed data analysis using GraphPad Prism 9 (GraphPad Software, La Jolla, CA, USA). For assessing overall survival in PDAC patients, the Kaplan–Meier method and log-rank test were applied for significance testing. Differences among groups were assessed using unpaired two-tailed Student’s t-tests (for two groups) or ANOVA (for three or more groups). Results are presented as mean ± SD, and a p-value less than 0.05 was considered statistically significant.

## Supplementary Material

1

2

3

4

5

6

7

8

## Figures and Tables

**Table:** KEY RESOURCES TABLE

REAGENT or RESOURCE	SOURCE	IDENTIFIER
Antibodies		
Mouse monoclonal anti-SERPINB3	R & D Systems	Cat# MAB6528; RRID:AB_2943627
Rabbit polyclonal anti-SERPINB3	Thermo Fisher Scientific	Cat# PA5-30164; RRID:AB_2547638
Mouse monoclonal anti-c-Myc	MilliporeSigma	Cat# M4439; RRID:AB_439694
Mouse monoclonal anti-BBOX1	MilliporeSigma	Cat# WH0008424M1; RRID:AB_1840194
Mouse monoclonal anti-β-actin	MilliporeSigma	Cat# A5441; RRID:AB_476744
Mouse monoclonal anti-Cas9	Cell Signaling Technology, Inc.	Cat# 14697; RRID:AB_2750916
Rabbit polyclonal anti-SERPINB3	Abcam	Cat# ab154971; RRID:AB_2943626
Rabbit polyclonal anti-CD31	Abcam	Cat# ab28364; RRID:AB_726362
Mouse monoclonal anti-8-hydroxy-2’-deoxyguanosine	Abcam	Cat# ab48508; RRID:AB_867461
Bacterial and virus strains		
DH5α Competent Cells for Subcloning	Thermo Fisher Scientific	Cat# EC0111
Biological samples		
Pancreatic tumor tissues from resected PDAC patients	University of Maryland Medical System (UMMS) in Baltimore, MD University Medical Center Göttingen, Germany	N/A
Chemicals, peptides, and recombinant proteins		
L-carnitine	MilliporeSigma	Cat# C0158
Hydroxyproline	MilliporeSigma	Cat# H5534
Gemcitabine hydrochloride	MilliporeSigma	Cat# G6423
Palmitic acid	MilliporeSigma	Cat# P5585
Bovine Serum Albumin	MilliporeSigma	Cat# A7030
cOmplete^™^ Protease Inhibitor Cocktail	MilliporeSigma	Cat# 4693116001
Calpeptin	MedChemExpress LLC	Cat# HY-100223
RPMI 1640 Medium, GlutaMAX^™^ Supplement	Thermo Fisher Scientific	Cat# 61870127
IMDM	Thermo Fisher Scientific	Cat# 12440053
McCoy’s 5A (Modified) Medium	Thermo Fisher Scientific	Cat# 16600082
RIPA Lysis and Extraction Buffer	Thermo Fisher Scientific	Cat# 89901
SuperBlock^™^ Blocking Buffer	Thermo Fisher Scientific	Cat# 37515
SuperSignal^™^ West Dura Extended Duration Substrate	Thermo Fisher Scientific	Cat# 34075
TRIzol^™^ Reagent	Thermo Fisher Scientific	Cat# 15596018
High-Capacity cDNA Reverse Transcription Kit	Thermo Fisher Scientific	Cat# 4368813
TaqMan^™^ Fast Advanced Master Mix for qPCR	Thermo Fisher Scientific	Cat# 4444557
Puromycin Dihydrochloride	Thermo Fisher Scientific	Cat# A1113803
Corning^®^ Matrigel^®^ Basement Membrane Matrix, LDEV-free	Corning	Cat# 354234
Target Retrieval Solution, pH 9	Agilent Technologies	Cat# S2367
Liquid DAB+	Agilent Technologies	Cat# K346811-2
RNeasy Micro Kit	QIAGEN	Cat# 74004
RNeasy Plus Mini Kit	QIAGEN	Cat# 74134
QIAfilter Plasmid Midi Kit	QIAGEN	Cat# 12243
DNeasy Blood & Tissue Kit	QIAGEN	Cat# 69506
Critical commercial assays		
Calpain-Glo^™^ Protease Assay	Promega	Cat# G8502
Cell Counting Kit-8	Dojindo	Cat# CK04
Pierce^™^ BCA Protein Assay Kits	Thermo Fisher Scientific	Cat# 23225
CellROX^™^ Deep Red	Thermo Fisher Scientific	Cat# C10422
MitoTracker^™^ Red FM	Thermo Fisher Scientific	Cat# M22425
MitoSOX^™^ Red	Thermo Fisher Scientific	Cat# M36008
Deposited data		
Raw RNA sequencing data	This paper	NCBI GEO: GSE223909. This SuperSeries is composed of the following SubSeries GSE224564; GSE223908; GSE223536
Raw metabolome data	This paper	https://osf.io/3jyut/?view_only=fed315a5a79f4cc283790eefc73fca3f
Experimental models: Cell lines		
AsPC-1	American type culture collection (ATCC)	RRID:CVCL_0152
BxPC-3	American type culture collection (ATCC)	RRID:CVCL_0186
Capan-1	American type culture collection (ATCC)	RRID:CVCL_0237
Capan-2	American type culture collection (ATCC)	RRID:CVCL_0026
CFPAC-1	American type culture collection (ATCC)	RRID:CVCL_1119
MIA PaCa-2	American type culture collection (ATCC)	RRID:CVCL_0428
PANC-1	American type culture collection (ATCC)	RRID:CVCL_0480
Panc 10.05	American type culture collection (ATCC)	RRID:CVCL_1639
SU.86.86	American type culture collection (ATCC)	RRID:CVCL_3881
293T	American type culture collection (ATCC)	RRID:CVCL_0063
Experimental models: Organisms/strains		
NOD-SCID mouse	Jackson Laboratory	RRID:IMSR_JAX:005557
Oligonucleotides		
All the oligonucleotides can be found in Table S7	This paper	N/A
Recombinant DNA		
Empty vector control	Genecopoeia	Cat# EX-NEG-Lv103
SERPINB3 vector	Genecopoeia	Cat# EX-F0390-Lv103
Software and algorithms		
MetaboAnalyst 5.0	Pang et al.^53^	RRID:SCR_015539
GraphPad Prism 9	GraphPad Software	RRID:SCR_002798
CFX384 Touch Real-Time PCR Detection System	Bio-Rad Laboratories, Inc.	RRID:SCR_018057
FlowJo version 10.8.1	Becton, Dickinson and Company	RRID:SCR_008520
Integrated Genomics Viewer (IGV)	Robinson et al.^54^	RRID:SCR_011793
Ingenuity Pathway Analysis	QIAGEN	RRID:SCR_008653
Partek Genomics Suite	Partek Inc.	RRID:SCR_011860

## Highlights

- SERPINB3 is linked to poor patient survival in pancreatic ductal adenocarcinoma (PDAC)
- SERPINB3 enhances tumor metastasis in an orthotopic preclinical PDAC mouse model
- SERPINB3-MYC axis induces the aggressive basal-like/squamous molecular subtype
- SERPINB3-MYC axis increases carnitine/acylcarnitine and amino acid metabolism
