# Supplementary material for: SERPINB3-MYC axis induces the basal-like/squamous subtype and enhances disease progression in pancreatic cancer
Source: Cell Rep. Author manuscript; Available in PMC 2024 Feb 5. (PMC10842852; doi:10.1016/j.celrep.2023.113434)
Supplement: 1 [file NIHMS1954755-supplement-1.pdf]

Cell Reports, Volume *42*

Supplemental information

SERPINB3-MYC axis induces the basal-like/squamous subtype and enhances disease progression in pancreatic cancer

Yuuki Ohara, Wei Tang, Huaitian Liu, Shouhui Yang, Tiffany H. Dorsey, Helen Cawley, Paloma Moreno, Raj Chari, Mary R. Guest, Azadeh Azizian, Jochen Gaedcke, Michael Ghadimi, Nader Hanna, Stefan Ambs, and S. Perwez Hussain

Figure S1

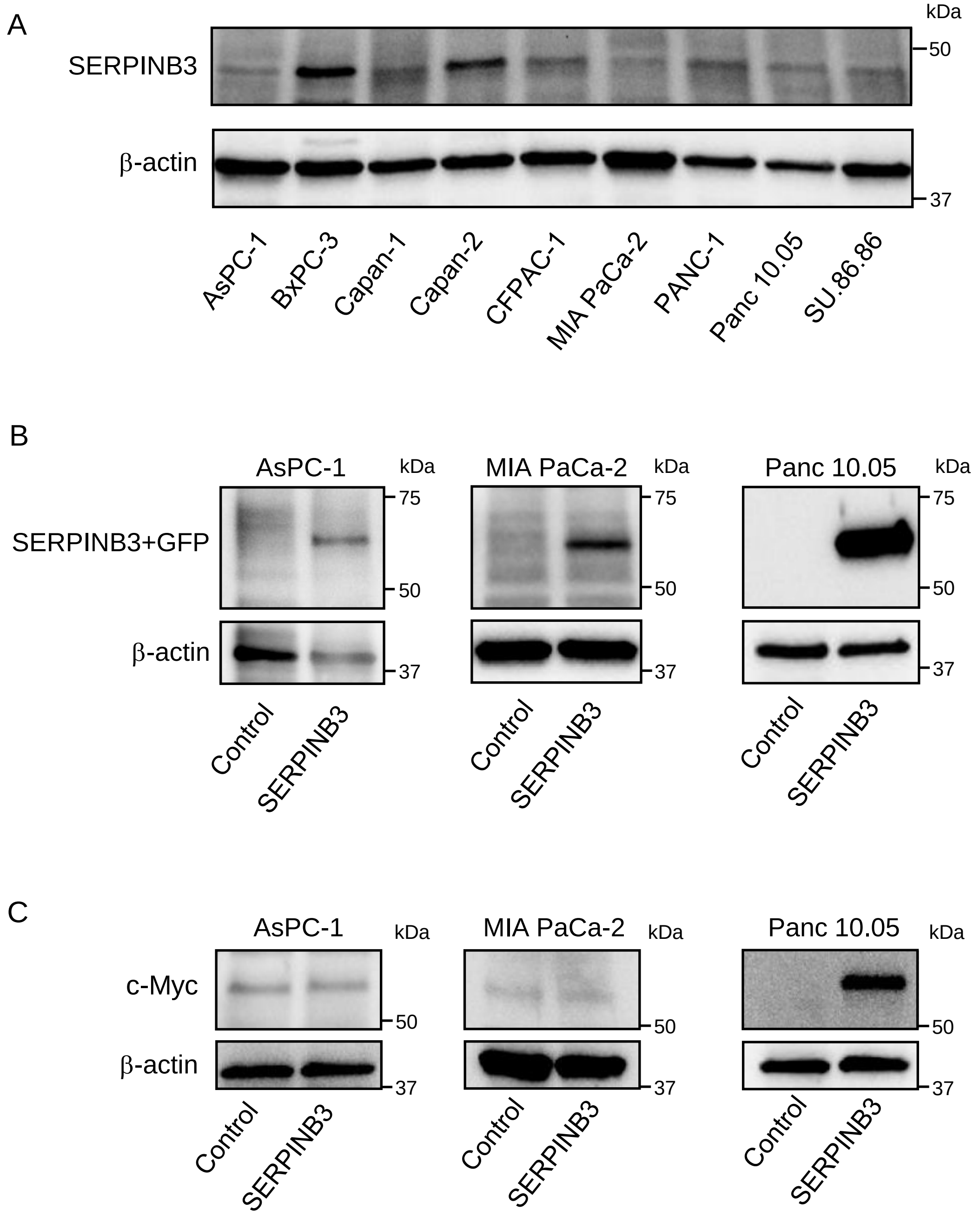
A
kDa
SERPINB3
50
β-actin
37
AsPC-1
BxPC-3
Capan-1
Capan-2
CFPAC-1
MIA PaCa-2
PANC-1
Panc 10.05
SU.86.86
B
AsPC-1
MIA PaCa-2
Panc 10.05
kDa
75
50
37
SERPINB3+GFP
β-actin
Control
SERPINB3
C
c-Myc
β-actin
Control
SERPINB3

Figure S1. Expression of SERPINB3 and c-Myc in human PDAC cell lines. Related to Figure 2.

(A) Endogenous SERPINB3 protein levels in human PDAC cell lines. AsPC-1, MIA PaCa-2, and Panc 10.05 cells were selected to establish cell lines with *SERPINB3* transgene expression. These cell lines are of the classical/progenitor PDAC subtype[1] and can be used to investigate the transition into the basal-like/squamous subtype following SERPINB3 overexpression.

(B) Confirmation of *SERPINB3* transgene overexpression at the protein level in AsPC-1, MIA PaCa-2, and Panc 10.05 cells.

(C) Examination of c-Myc protein levels in PDAC cell lines overexpressing SERPINB3. While AsPC-1 and MIA PaCa-2 cells did not show c-Myc upregulation at the protein level, Panc 10.05 cells exhibited a significant upregulation of c-Myc protein.

Figure S2

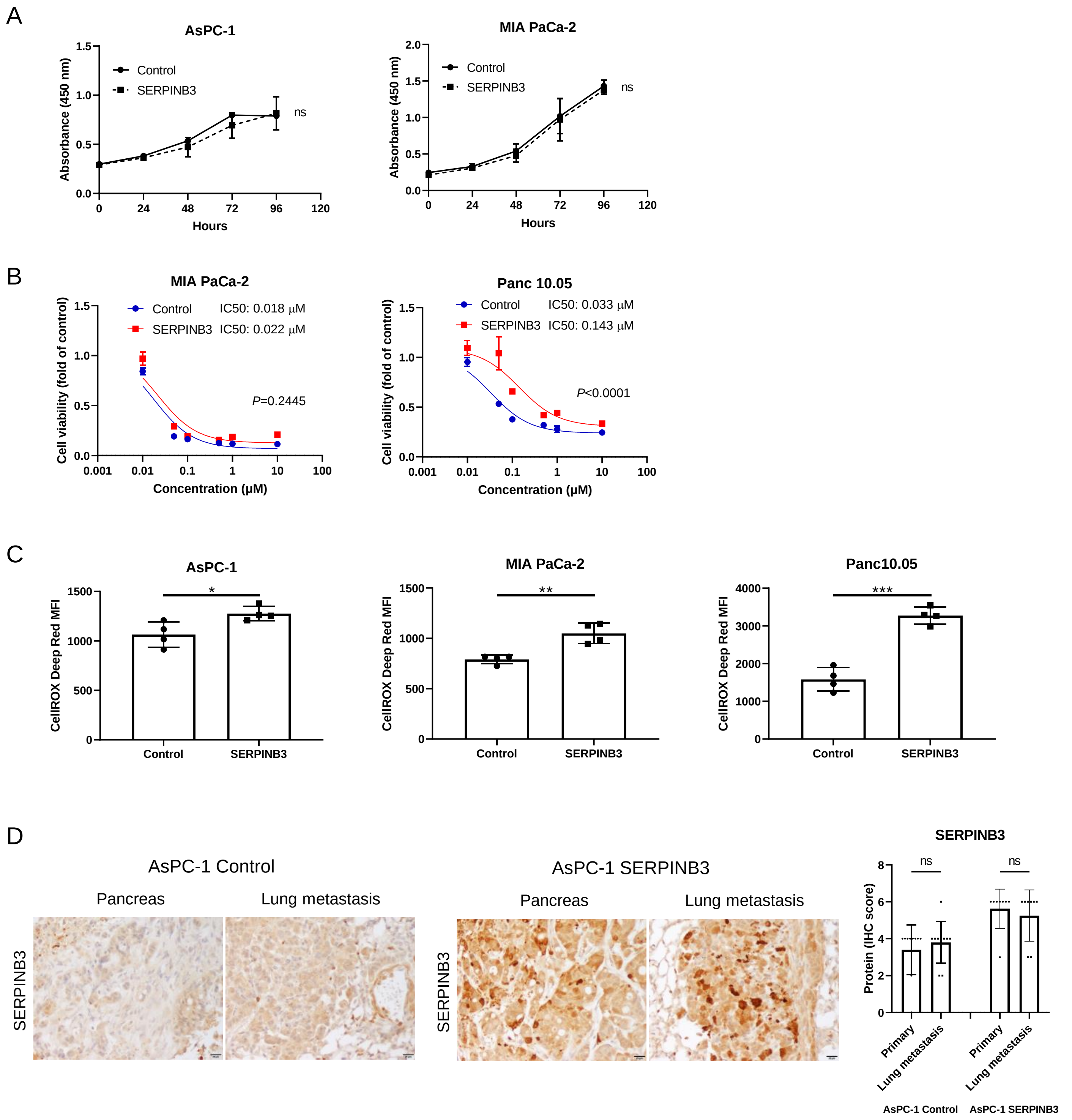
A
AsPC-1
Absorbance (450 nm)
Control
SERPINB3
ns
Hours
MIA PaCa-2
B
MIA PaCa-2
Cell viability (fold of control)
Control IC50: 0.018 µM
SERPINB3 IC50: 0.022 µM
P=0.2445
Concentration (µM)
Panc 10.05
Control IC50: 0.033 µM
SERPINB3 IC50: 0.143 µM
P<0.0001
C
AsPC-1
MIA PaCa-2
Panc10.05
CellROX Deep Red MFI
*
**
***
Control
SERPINB3
D
AsPC-1 Control
AsPC-1 SERPINB3
Pancreas
Lung metastasis
SERPINB3
Protein (IHC score)
ns
Primary
Lung metastasis
AsPC-1 Control
AsPC-1 SERPINB3

Figure S2. The impact of SERPINB3 on proliferation, gemcitabine sensitivity, oxidative stress, and metastasis in PDAC cells. Related to Figure 3.

SERPINB3 transgene expressing PDAC cells were examined to define the function of SERPINB3.

(A) SERPINB3 overexpression did not enhance the proliferation of PDAC cell lines (CCK-8/WST-8 assay). Data are presented as the mean ± SD of three independent experiments; ns (not significant) by two-way ANOVA.

(B) Dose-response curves showing viability of gemcitabine-treated PDAC cell lines (CCK-8/WST-8 assay). SERPINB3 reduces gemcitabine sensitivity in Panc 10.05 but not in MIA PaCa-2 cells. Data are presented as the mean ± SD (n = 3 for each group).

(C) Levels of reactive oxygen species in the SERPINB3-overexpressing PDAC cells and the control PDAC cells, showing that SERPINB3 promotes oxidative stress levels. Data are presented as the mean ± SD (n = 4 for each group); *$p < 0.05$, **$p < 0.01$, ***$p < 0.005$ by unpaired two-tailed Student's t-test.

(D) Persistence of SERPINB3 expression in the lung metastatic sites. IHC for SERPINB3 was performed in the primary PDAC xenografts and the resulting lung metastases of the PDAC xenograft model. The expression of SERPINB3 remained preserved in the lung metastatic sites, comparable to the primary lesion, in both the control group and the SERPINB3-expressing group. Scale bar is 20 μm. Data are presented as the mean ± SD (AsPC-1 control; n = 10 mice, AsPC-1 SERPINB3; n = 8 mice); ns (not significant) by unpaired two-tailed Student's t-test.

IHC; immunohistochemistry.

Figure S3

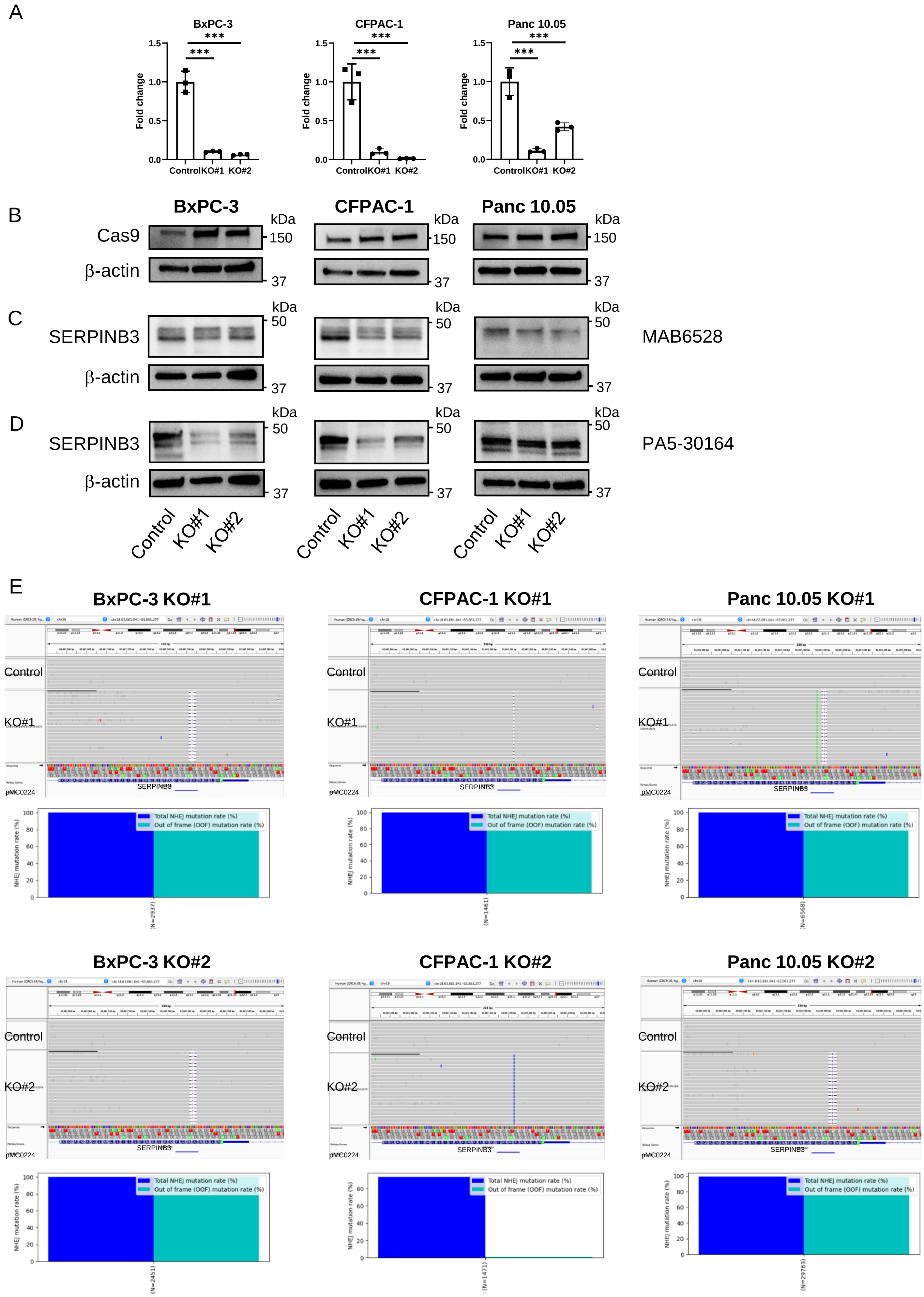
A
BxPC-3
CFPAC-1
Panc 10.05
***
Fold change
1.5
1.0
0.5
0.0
Control
KO#1
KO#2
B
Cas9
β-actin
kDa
150
37
C
SERPINB3
50
MAB6528
D
PA5-30164
E
BxPC-3 KO#1
CFPAC-1 KO#1
Panc 10.05 KO#1
BxPC-3 KO#2
CFPAC-1 KO#2
Panc 10.05 KO#2
SERPINB3
pMC0224
Total NHEJ mutation rate (%)
Out of frame (OOF) mutation rate (%)
NHEJ mutation rate (%)
(N=2937)
(N=1461)
(N=6568)
(N=2451)
(N=1471)
(N=29763)

Figure S3. Establishment and verification of SERPINB3 knockout in human PDAC cell lines. Related to Figure 4.

(A) Quantitative real-time PCR analysis indicates a significant approximate 90% decrease in *SERPINB3* expression within the SERPINB3-knockout PDAC cell lines. Data are presented as the mean ± SD of three independent experiments; ***$p < 0.005$ by one-way ANOVA.

(B) Verification of Cas9 protein expression was established in the SERPINB3-knockout PDAC cell lines.

(C and D) Validation of SERPINB3 protein reduction in the SERPINB3-knockout PDAC cell lines using two distinct anti-SERPINB3 antibodies.

(E) Assessment of *SERPINB3* DNA editing was performed using MiSeq, confirming 100% editing efficiency for both NHEJ and out of frame mutations in the SERPINB3-knockout PDAC cell lines (BxPC-3 KO#1, BxPC-3 KO#2, CFPAC-1 KO#1, Panc 10.05 KO#1, and Panc 10.05 KO#2).

The residual presence of the bands around 45 kDa in the western blot (C and D) may be attributed to potential cross-reactivity with proteins of similar molecular weight.

Figure S4

A

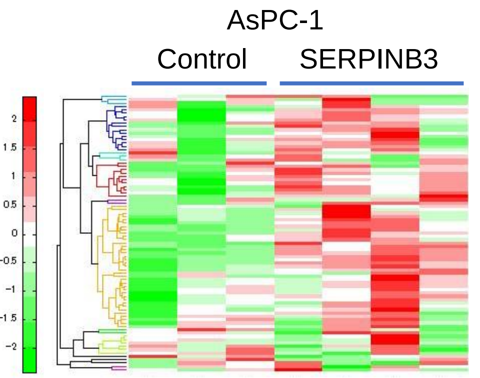

20 metabolites↑
0 metabolites↓

B Amino acids

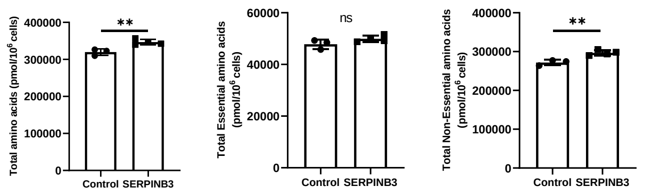

C

Pathways	*P* value
Glutamate Metabolism	7.81E-07
Glycolysis	2.47E-06
Warburg Effect	3.03E-06
Urea Cycle	6.33E-06
Ammonia Recycling	1.17E-05
Gluconeogenesis	2.02E-05
Glycine and Serine Metabolism	4.32E-05
Folate Metabolism	1.13E-04
Alanine Metabolism	1.74E-04
Purine Metabolism	1.94E-04
Amino Sugar Metabolism	2.15E-04
Arginine and Proline Metabolism	2.34E-04
Butyrate Metabolism	2.76E-04
Glutathione Metabolism	4.16E-04
Valine, Leucine and Isoleucine Degradation	4.70E-04
Transfer of Acetyl Groups into Mitochondria	5.03E-04
Malate-Aspartate Shuttle	6.00E-04
Propanoate Metabolism	6.93E-04

D Upstream analysis using the 20 metabolites

Upstream Regulator	z-score	*P* value
SIX1	2.236	4.27E-05
AQP7	2.200	3.49E-06
glutamine	2.179	1.91E-06
→ MYC	1.982	1.25E-03
D-glucose	1.474	4.64E-08

E

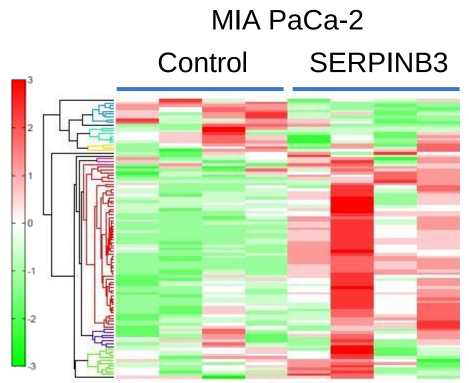

36 metabolites↑
5 metabolites↓

F Amino acids

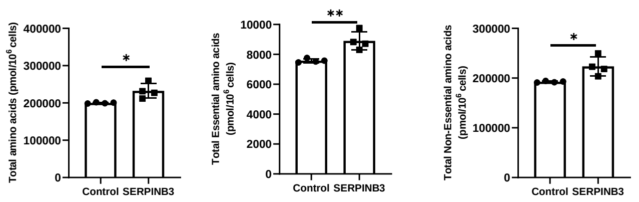

G

	P value
Warburg Effect	1.06E-07
Glutamate Metabolism	1.69E-07
Ammonia Recycling	3.50E-07
Glycine and Serine Metabolism	1.30E-06
Citric Acid Cycle	4.76E-06
Methionine Metabolism	5.42E-06
Alanine Metabolism	9.70E-06
Gluconeogenesis	9.91E-06
Purine Metabolism	1.37E-05
Pyruvate Metabolism	1.43E-05
Pentose Phosphate Pathway	2.67E-05
Urea Cycle	2.67E-05
Transfer of Acetyl Groups into Mitochondria	5.16E-05
Aspartate Metabolism	9.91E-05
Selenoamino Acid Metabolism	2.25E-04
Arginine and Proline Metabolism	2.41E-04
⋮	⋮
→ Carnitine Synthesis	3.61E-02

H Upstream analysis using the 36 metabolites

Upstream Regulator	z-score	*P* value
CA9	3.464	1.30E-17
→ MYC	3.000	1.44E-07
SIX1	2.333	7.38E-10
HNF1B	2.236	2.71E-05
lipopolysaccharide	2.236	8.45E-03
HDAC11	2.200	1.53E-07
GCG	2.186	1.25E-05
D-glucose	2.084	1.39E-07

Figure S4. Upregulation of amino acid and carnitine metabolism in SERPINB3-overexpressing PDAC cells. Related to Figure 6, Table S5, and Table S6.

(A and B) Heatmap summarizing a metabolome analysis of 116 cancer-related metabolites in SERPINB3-overexpressing AsPC-1 cells, comparing their levels with vector control cells. In SERPINB3-overexpressing AsPC-1 cells, 20 metabolites were significantly elevated when compared with control cells ($p < 0.05$). The graphs illustrate the increase in amino acid metabolism. Red = upregulated; green = downregulated. Data are presented as the mean ± SD (AsPC-1 control; n = 3, AsPC-1 SERPINB3; n = 4); **$p < 0.01$, ns (not significant) by unpaired two-tailed Student's t-test.

(C) Pathway enrichment scores using MetaboAnalyst 5.0 with 20 input metabolites, indicating that amino acid metabolism and the Warburg effect were activated in SERPINB3-overexpressing AsPC-1 cells.

(D) Prediction of upstream regulators for the metabolism in SERPINB3-overexpressing AsPC-1 cells. The upstream analysis by IPA using the 20 input metabolites predicts upregulated MYC signaling in these cells.

(E and F) Heatmap summarizing a metabolome analysis of 116 cancer-related metabolites in SERPINB3-overexpressing MIA PaCa-2 cells, comparing their levels with vector control cells. In SERPINB3-overexpressing MIA PaCa-2 cells, 36 metabolites were significantly elevated and 5 were decreased when compared with control cells ($p < 0.05$). The graphs illustrate the increase in amino acid metabolism. Data are presented as the mean ± SD (n = 4 for each group); *$p < 0.05$, **$p < 0.01$ by unpaired two-tailed Student's t-test.

(G) Pathway enrichment scores using MetaboAnalyst 5.0 with 36 input metabolites, indicating that amino acid and carnitine metabolism and the Warburg effect were activated in SERPINB3-overexpressing MIA PaCa-2 cells.

(H) Prediction of upstream regulators for the metabolism in SERPINB3-overexpressing MIA PaCa-2 cells. The upstream analysis by IPA using the 36 input metabolites predicts upregulated MYC signaling in these cells.

IPA; Ingenuity pathway analysis.

Figure S5

A

CFPAC-1

Control SERPINB3KO

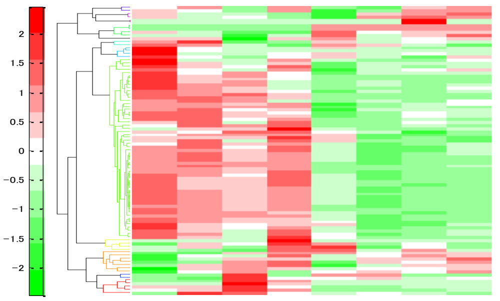

3 metabolites↑
54 metabolites↓

B Amino acids

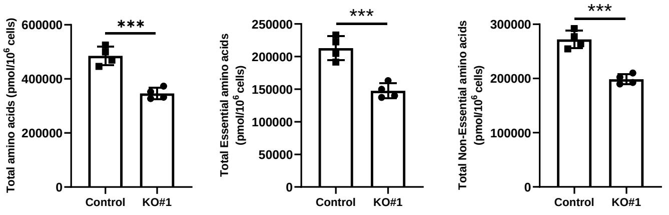

C

Pathways	*P* value
Warburg Effect	2.23E-09
Purine Metabolism	1.39E-08
Glutamate Metabolism	1.74E-08
Aspartate Metabolism	2.20E-08
Arginine and Proline Metabolism	4.68E-07
Citric Acid Cycle	1.08E-06
Transfer of Acetyl Groups into Mitochondria	4.07E-06
Urea Cycle	4.32E-06
Ammonia Recycling	1.08E-05
Beta-Alanine Metabolism	1.87E-05
Histidine Metabolism	2.11E-05
Pyruvate Metabolism	5.93E-05
Valine, Leucine and Isoleucine Degradation	8.46E-05
Phytanic Acid Peroxisomal Oxidation	1.59E-04
Mitochondrial Electron Transport Chain	1.87E-04
Nicotinate and Nicotinamide Metabolism	2.76E-04
⋮	⋮
→ Carnitine Synthesis	2.05E-02

D

Upstream analysis using the 54 metabolites

Upstream Regulator	z-score	*P* value
CA9	-3.500	3.83E-25
SIX1	-3.317	2.45E-10
⋮	⋮	⋮
→ MYC	-2.891	2.74E-10

E

Panc 10.05

Control SERPINB3KO

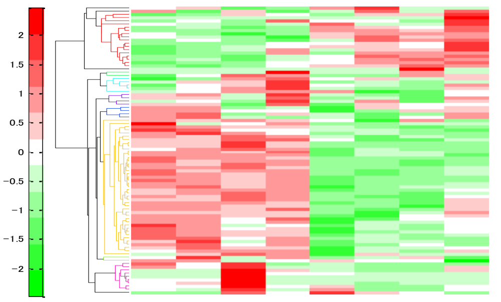

11 metabolites↑
36 metabolites↓

F Amino acids

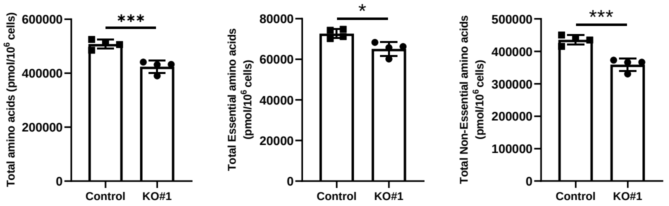

G

Pathways	*P* value
Warburg Effect	5.46E-07
Urea Cycle	1.76E-05
Arginine and Proline Metabolism	1.99E-05
Glycine and Serine Metabolism	4.93E-05
Valine, Leucine and Isoleucine Degradation	5.67E-05
Aspartate Metabolism	6.62E-05
Glutamate Metabolism	8.57E-05
Butyrate Metabolism	2.15E-04
Ammonia Recycling	3.49E-04
Ketone Body Metabolism	5.22E-04
Gluconeogenesis	5.83E-04
Nicotinate and Nicotinamide Metabolism	7.97E-04
Propanoate Metabolism	1.60E-03
Purine Metabolism	1.62E-03
Histidine Metabolism	1.82E-03
Phenylacetate Metabolism	2.25E-03
⋮	3.63E-03
→ Carnitine Synthesis	4.35E-03

H

Upstream analysis using the 36 metabolites

Upstream Regulator	z-score	*P* value
CD40	-3.162	7.79E-11
CA9	-2.887	8.23E-19
⋮	⋮	⋮
→ MYC	-1.808	4.15E-07

Figure S5. Downregulation of amino acid and carnitine metabolism in SERPINB3-knockout PDAC cells. Related to Figure 6, Table S5, and Table S6.

(A and B) Metabolome analysis covering 116 cancer-related metabolites in SERPINB3-knockout CFPAC-1 cells. In SERPINB3-knockout CFPAC-1 cells, 54 metabolites were significantly downregulated when compared with control cells ($p < 0.05$). The graphs illustrate the decrease in amino acid metabolism. Data are presented as the mean ± SD (n = 4 for each group); ***p < 0.005 by unpaired two-tailed Student's t-test.

(C) Pathway enrichment scores using MetaboAnalyst 5.0 with the significantly decreased 54 input metabolites, indicating that amino acid and carnitine metabolism and the Warburg effect were downregulated in SERPINB3-knockout CFPAC-1 cells.

(D) Prediction of upstream regulators for the metabolism in SERPINB3-knockout CFPAC-1 cells. The upstream analysis by IPA using the 54 input metabolites predicts downregulated MYC signaling in these cells.

(E and F) Metabolome analysis covering 116 cancer-related metabolites in SERPINB3-knockout Panc 10.05 cells. In SERPINB3-knockout Panc 10.05 cells, 36 metabolites were significantly downregulated when compared with control cells ($p < 0.05$). The graphs illustrate the decrease in amino acid metabolism. Data are presented as the mean ± SD (n = 4 for each group); *p < 0.05, ***p < 0.005 by unpaired two-tailed Student's t-test.

(G) Pathway enrichment scores using MetaboAnalyst 5.0 with significantly downregulated 36 input metabolites, indicating that amino acid and carnitine metabolism and the Warburg effect were downregulated in SERPINB3-knockout Panc 10.05 cells.

(H) Prediction of upstream regulators for the metabolism in SERPINB3-knockout Panc 10.05 cells. The upstream analysis by IPA using the 36 input metabolites predicts downregulated MYC signaling in these cells.

IPA; Ingenuity pathway analysis.

Figure S6

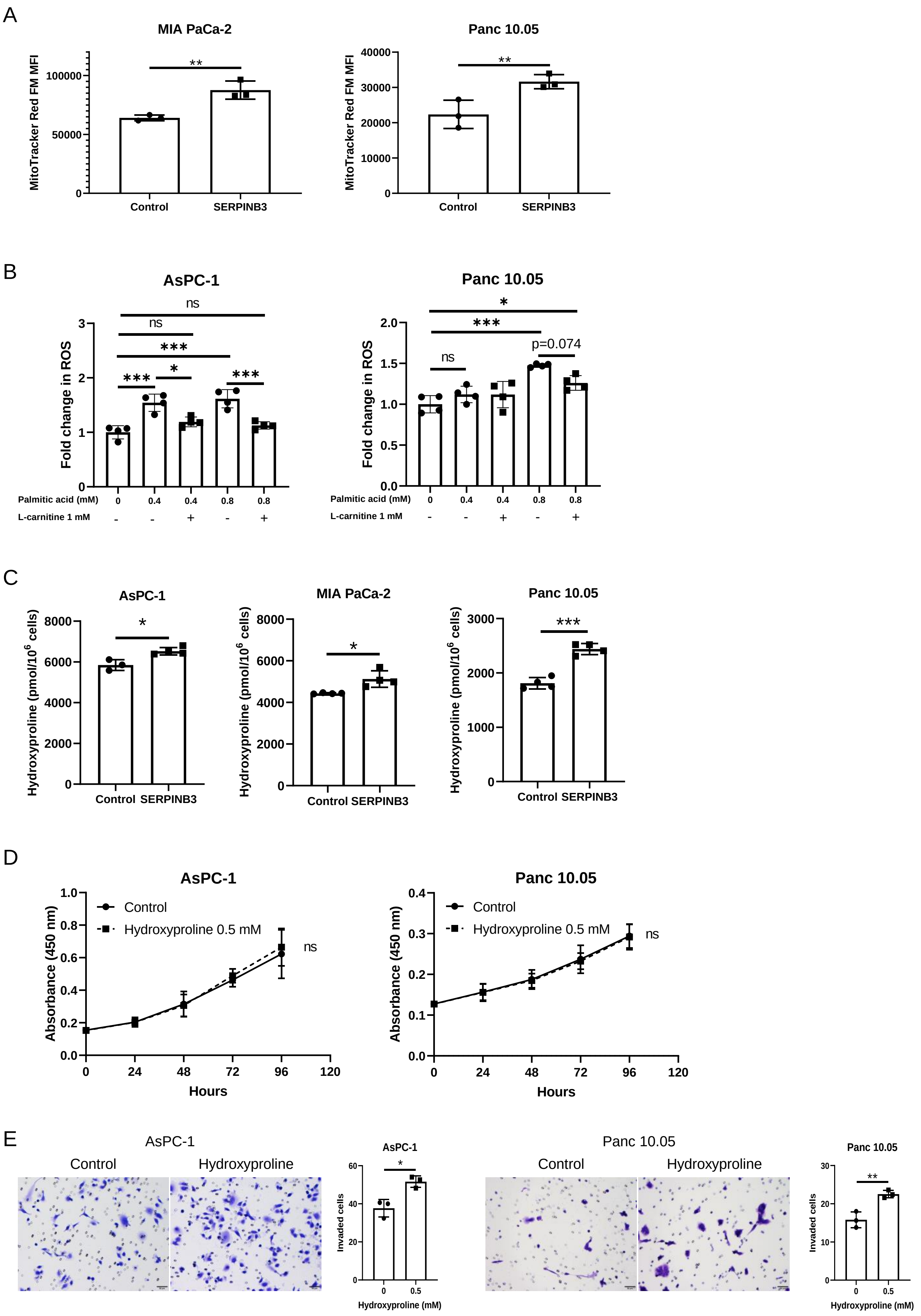
A
MIA PaCa-2
Panc 10.05
MitoTracker Red FM MFI
Control
SERPINB3
**
B
AsPC-1
Panc 10.05
Fold change in ROS
Palmitic acid (mM)
L-carnitine 1 mM
ns
***
*
p=0.074
C
AsPC-1
MIA PaCa-2
Panc 10.05
Hydroxyproline (pmol/10^6 cells)
Control
SERPINB3
D
AsPC-1
Panc 10.05
Control
Hydroxyproline 0.5 mM
Absorbance (450 nm)
Hours
ns
E
AsPC-1
Panc 10.05
Control
Hydroxyproline
Invaded cells
Hydroxyproline (mM)

Figure S6. SERPINB3-associated metabolic effects in human PDAC cell progression. Related to Figure 7.

(A) Increased mitochondrial membrane potential in SERPINB3-overexpressing PDAC cells. The mitochondrial membrane potential was quantified in cells using MitoTracker™ Red FM and flow cytometry. Data are presented as the mean ± SD (n = 3 for each group); $^{**}p < 0.01$ by unpaired two-tailed Student's t-test.

(B) L-carnitine supplementation reduced oxidative stress induced by palmitic acid in PDAC cells. Mitochondrial reactive oxygen species (ROS) were quantified using MitoSOX™ Red and a microplate reader. Data are presented as the mean ± SD (n = 4 for each group); $^{*}p < 0.05$, $^{***}p < 0.005$, ns (not significant) by one-way ANOVA.

(C) Increased hydroxyproline level in SERPINB3-overexpressing PDAC cells. Data are presented as the mean ± SD (AsPC-1 control; n = 3, AsPC-1 SERPINB3; n = 4, MIA PaCa-2 control; n = 4, MIA PaCa-2 SERPINB3; n = 4, Panc 10.05 control; n = 4, Panc 10.05 SERPINB3; n = 4); $^{*}p < 0.05$, $^{***}p < 0.005$, ns (not significant) by unpaired two-tailed Student's t-test.

(D) Hydroxyproline supplementation in the culture medium did not exert a significant effect on proliferation (CCK-8/WST-8 assay). Data are presented as the mean ± SD of three independent experiments; ns (not significant) by two-way ANOVA.

(E) Hydroxyproline increased invasiveness of PDAC cell lines. Cells that passed through a cell culture insert membrane coated with Matrigel were fixed, and the cells were counted. Data are presented as the mean ± SD of three independent experiments; $^{*}p < 0.05$, $^{**}p < 0.01$ by unpaired two-tailed Student's t-test.

Figure S7

NCI-UMD-German cohort

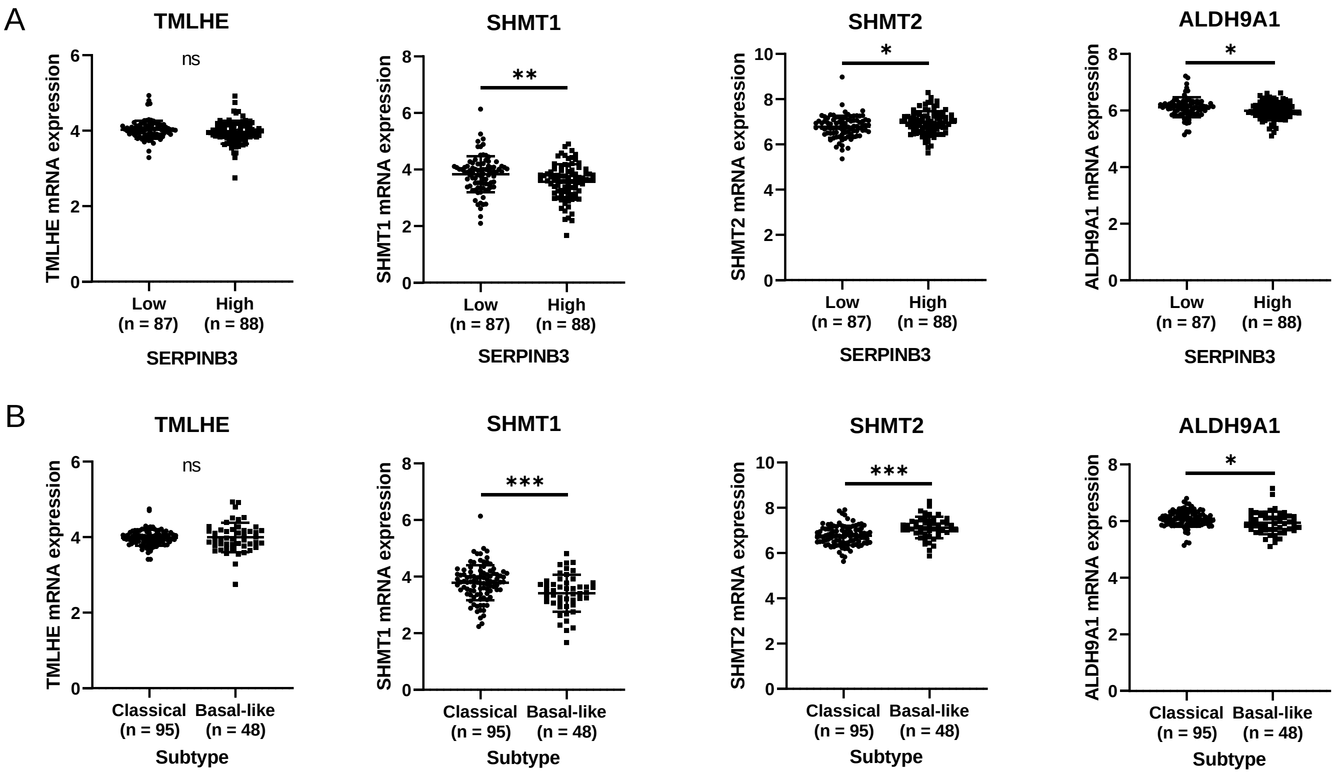

PDAC cell lines

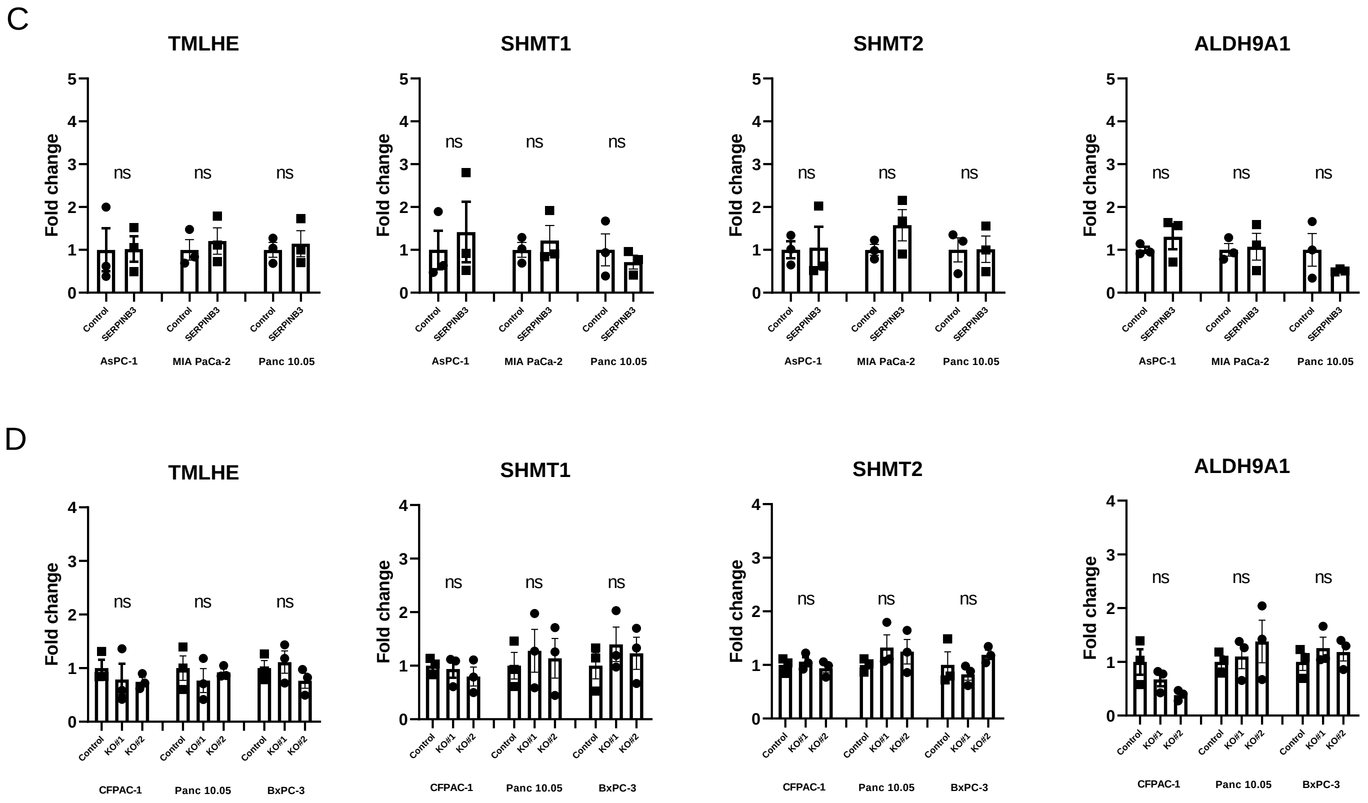

Figure S7. A comprehensive analysis of genes implicated in L-carnitine synthesis in PDAC. Related to Figure 7.

(A and B) Expression of L-carnitine synthesis genes in clinical PDAC samples from NCI-UMD-German cohort. Data are presented as the mean ± SD; $*p < 0.05$, $**p < 0.01$, $***p < 0.005$, ns (not significant) by unpaired two-tailed Student's t-test.

(C and D) Expression of L-carnitine synthesis genes in human PDAC cell lines. Data are presented as the mean ± SD of three independent experiments; ns (not significant) by unpaired two-tailed Student's t-test.

Supplemental References

1. Yu, K., Chen, B., Aran, D., Charalel, J., Yau, C., Wolf, D.M., van ‘t Veer, L.J., Butte, A.J., Goldstein, T., and Sirota, M. (2019). Comprehensive transcriptomic analysis of cell lines as models of primary tumors across 22 tumor types. Nature Communications *10*, 3574. 10.1038/s41467-019-11415-2.
